# Are there choices in the darkness? habitat selection and environmental filtering shape invertebrate communities in semi-arid caves

**DOI:** 10.1007/s00442-026-05940-3

**Published:** 2026-07-30

**Authors:** Vitor Gabriel Pereira Junta, Marconi Souza-Silva, Rodrigo Lopes Ferreira

**Affiliations:** 1 ¹Centro de Estudos em Biologia Subterrânea, Departamento de Ecologia e Conservação, Instituto de Ciências Naturais, Universidade Federal de Lavras, Campus Universitário, P.O. Box 3037, Lavras, CEP 37200-000 Minas Gerais Brasil; 2 Programa de Pós-graduação em Ecologia Aplicada, Universidade Federal de Lavras, Lavras, CEP, 37200-000 Minas Gerais Brasil

**Keywords:** Cave biodiversity, Niche-based processes, Community structure, Invertebrate communities, Tropical karst

## Abstract

Habitat selection governs subterranean organism distributions, where environmental gradients and restricted resources impose strong constraints, suggesting many cave-dwelling species respond primarily to fine-scale microhabitat variation rather than broad spatial patterns. To evaluate this hypothesis, we examined environmental drivers of invertebrate richness and community composition across 24 limestone caves in a tropical semi-arid region. Adopting a multi-scale framework, we quantified landscape attributes, cave-level habitat features, and local microhabitat conditions, recording 336 invertebrate species representing 40 orders and 106 families, including 38 obligate subterranean taxa. Our findings demonstrate that these communities are governed by a hierarchy of localized environmental filters, while regional spatial processes exert a negligible footprint. At the mesoscale, cave identity and internal sectors heavily determine community composition, overriding geographic distance and maintaining high species turnover. Across internal gradients, non-troglobitic and troglobitic fauna display an inverse spatial sorting between scales. For non-troglobitic communities, species richness declines sharply toward deeper zones, a negative effect intensified at the microscale where these organisms respond positively to trophic resource availability. Conversely, obligate troglobitic richness is constrained by moisture availability at the mesoscale but becomes solely and positively driven by distance from the entrance at the microscale, as specialized species track deep, buffered microhabitats to avoid competitive interactions in high-energy zones. Together, our results confirm that niche-based environmental filtering structures these assemblages, positioning each cave as an ecologically singular unit. Consequently, Brazilian environmental licensing cannot rely on regional offsetting, since individual caves are not ecological substitutes, and fine-scale inventories are critical to prevent biodiversity loss.

**Supplementary Information:**

The online version contains supplementary material available at https://doi.org/10.1007/s00442-026-05940-3.

## Introduction

Environmental variability is a fundamental determinant of community organization, as distinct environmental attributes can generate contrasting biological responses and, in turn, produce heterogeneous patterns of species distribution (Tews et al. 2004; Odum and Barrett 2006). In this context, habitat heterogeneity has long been recognized as a major driver of biodiversity, because environments with greater structural and physicochemical complexity offer a wider spectrum of ecological opportunities, thereby promoting species coexistence (Stein et al. 2015; Pacheco et al. 2020; Souza-Silva et al. 2021). Within subterranean ecosystems, habitat heterogeneity plays an equally important role in shaping species distributions, despite the highly distinctive and constrained nature of underground environments.

Compared to surface environments, caves are generally characterized by marked climatic buffering, including limited annual temperature variation, persistently high humidity, permanent darkness beyond entrance zones, and chronically low levels of nutrient input (Howarth 1980, 1983; Culver and Pipan 2009). However, despite this apparent stability, caves exhibit pronounced internal environmental gradients. One of the most influential of these is the distance from the cave entrance, which governs both microclimatic conditions and the magnitude of organic matter inputs (Ficetola et al. 2018; Mammola 2019; Souza-Silva et al. 2021; Furtado et al. 2022). Because most trophic subsidies and many non-obligate subterranean taxa originate in epigean habitats and enter caves through their openings, increasing distance from the entrance is typically associated with declining resource availability and reduced species richness (Moseley 2008; Tobin et al. 2013; Ficetola et al. 2018; Mammola 2019). In parallel, caves display a clear zonation from environmentally variable entrance sectors to increasingly stable deep zones, generating strong spatial structuring of abiotic conditions within the subterranean environment (Moseley 2009; Tobin et al. 2013; Lunghi et al. 2014; Prous et al. 2015; Mammola and Isaia 2018; Lunghi and Manenti 2020; Souza-Silva et al. 2021).

This internal zonation results in a complex mosaic of microhabitats that differ in substrate characteristics, microclimatic regimes, and light availability, thereby enhancing fine-scale habitat heterogeneity within caves (Moseley 2008; Souza-Silva et al. 2011, 2021; Du Preez et al. 2015; Lunghi et al. 2017; Mammola and Isaia 2017; Mammola 2019; Lunghi and Manenti 2020; Mammola et al. 2020). Such environmental filtering favors the persistence of taxa that are pre-adapted to subterranean conditions, while simultaneously imposing strong selective pressures that can drive evolutionary specialization. Over evolutionary timescales, colonization of subterranean habitats has led to the emergence of obligate organisms (troglobionts) with specialized traits, narrow geographic ranges, and low population densities (Sket 2008; Culver and Pipan 2009). Consequently, these species exhibit a heightened sensitivity to microclimatic variations, rendering them particularly susceptible to environmental disturbances across different spatial scales (Mammola et al. 2019). As a result, caves may function both as long-term climatic refuges and as repositories preserving ancient and relict faunal lineages (Culver and Sket 2000; Sobral-Souza et al. 2015).

An ongoing discussion in subterranean ecology concerns whether regional connectivity across karst landscapes homogenizes biological communities or if localized environmental filters drive species turnover, making individual systems ecologically distinct (Rabelo et al. 2020; Pacheco et al. 2025). While multi-scale approaches are increasingly utilized to investigate these dynamics, empirical studies that integrate regional landscape attributes with within-cave environmental dimensions remain relatively limited (Bento et al. 2021; Souza-Silva et al. 2021; Furtado et al. 2022; Veiga et al. 2025). Furthermore, exploring whether individual caves tend to function as unique ecological units, and how specialized troglobites and facultative fauna might differ in their responses to these nested spatial scales, represents an open question. Particularly, these dynamics remain unclear in highly seasonal and climatically severe semi-arid landscapes where caves act as important evolutionary refuges.

Within this conceptual framework, the present study seeks to identify the habitat variables that regulate the richness and composition of cave-dwelling invertebrate communities. Specifically, we test the hypotheses that: (i) species richness declines along increasing gradients of distance from cave entrances; (ii) variation in community composition among different caves is non-random and primarily driven by cave-specific environmental characteristics; and (iii) invertebrate assemblages respond to habitat heterogeneity on the cave floor in a scale-dependent manner, varying across distinct spatial scales. Finally, we examine the implications of our findings for conservation, highlighting the role of this region as a significant reservoir of subterranean biodiversity.

## Materials and methods

### Study area

**Fig. 1:**
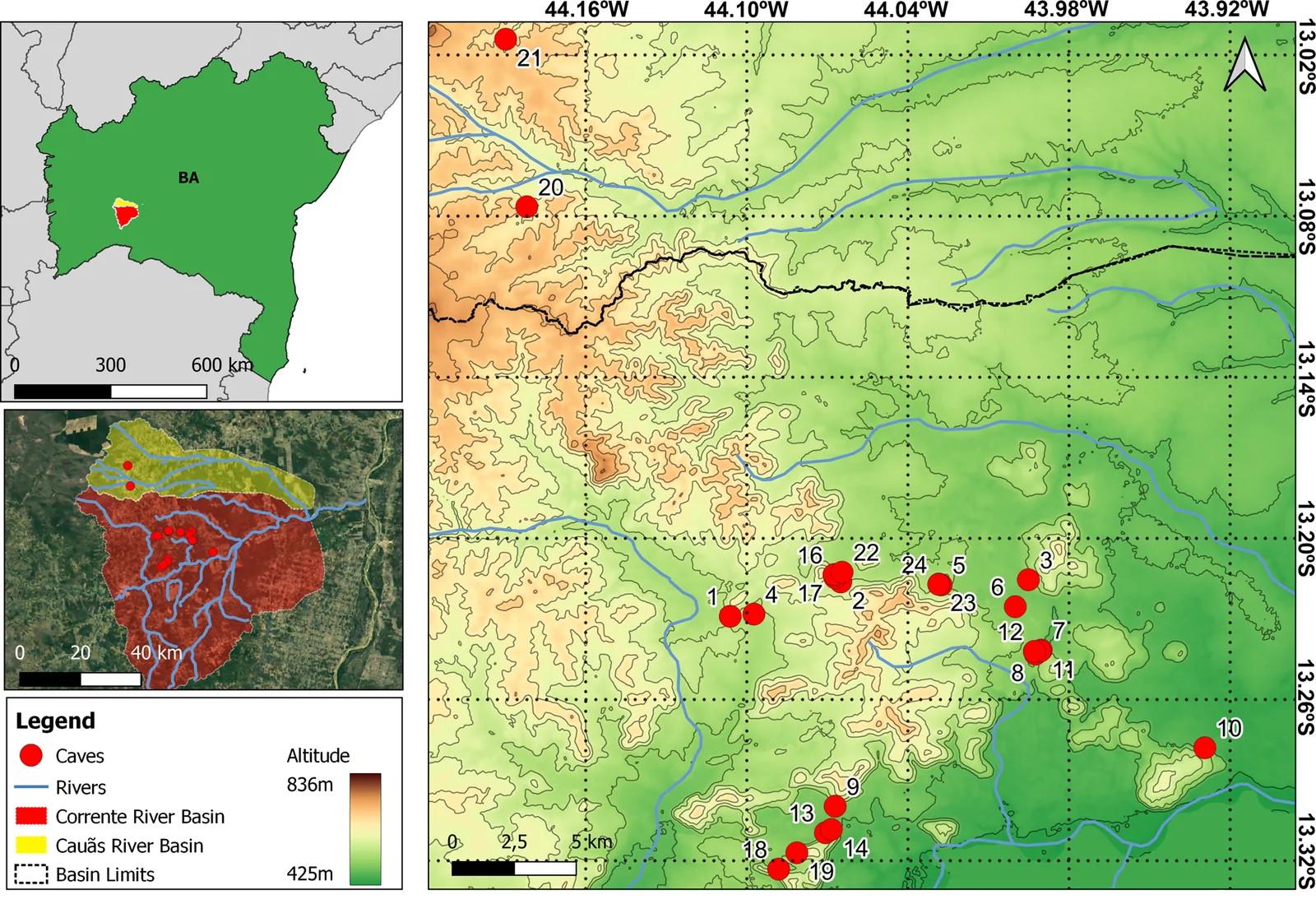
Location map of the caves sampled in the Santana region. BA – Bahia State; **1** – Cânion da Baixa Verde Cave; **2** – Padre Cave; **3** – Labirinto do Toxodon Cave; **4** – Boqueirão Cave; **5** – Pedra Escrevida Cave; **6** – Duas Cobras Cave; **7** – Tunel II Cave; **8** – São Geraldo Cave; **9** – Olho D’água do Cumbra Cave; **10** – Racha Bovina Cave; **11** – Tunel I Cave; **12** – Couve-Flor Cave; **13** – Geraldo Cruz Cave; **14** – Fenda Oblíqua Cave; **15** – Cedro Cave; **16** – Cedrão Cave; **17** – Cedrículo Cave; **18** – Pajeú Cave; **19** – Cristal Cave; **20** – Salobro Cave; **21** – Grota Cave; **22** – Leão Cave; **23** – Cinquentona Cave; **24** – Pedra Escrevidinha Cave

Field sampling was carried out in 24 limestone caves (Supplementary Material – Table S1) during two survey periods, from 23 August to 2 September 2021 and from 23 May to 2 June 2022, in the municipalities of Santana, Santa Maria da Vitória, and Canápolis, state of Bahia, Brazil (Fig. 1). The study area lies within an ecotonal zone between Seasonally Dry Tropical Forests and the Caatinga biome and is classified as Aw under the Köppen climate system, characterized by dry winters and rainy summers (Köppen 1936). This region is recognized for its high potential for endemism (Dinerstein 2017) and is situated within the Corrente River basin, one of the main tributaries of the São Francisco River (Fig. 1).

All sampling campaigns were restricted to the dry season for safety reasons. Some caves in the region are crossed by subterranean rivers with extensive catchment basins, which substantially increases the risk of sudden flooding during the rainy season, making cave access particularly hazardous. The study area is located within the Bambuí Limestone Group, the largest carbonate formation in Brazil, covering more than 145,000 km² and encompassing over 6,000 registered caves (Auler et al. 2019). Importantly, this region is recognized as a priority area for the conservation of Brazilian speleological heritage, according to the national mapping produced by the Centro Nacional de Pesquisa e Conservação de Cavernas (CECAV).

### Field procedures

#### Sampling design

**Fig. 2:**
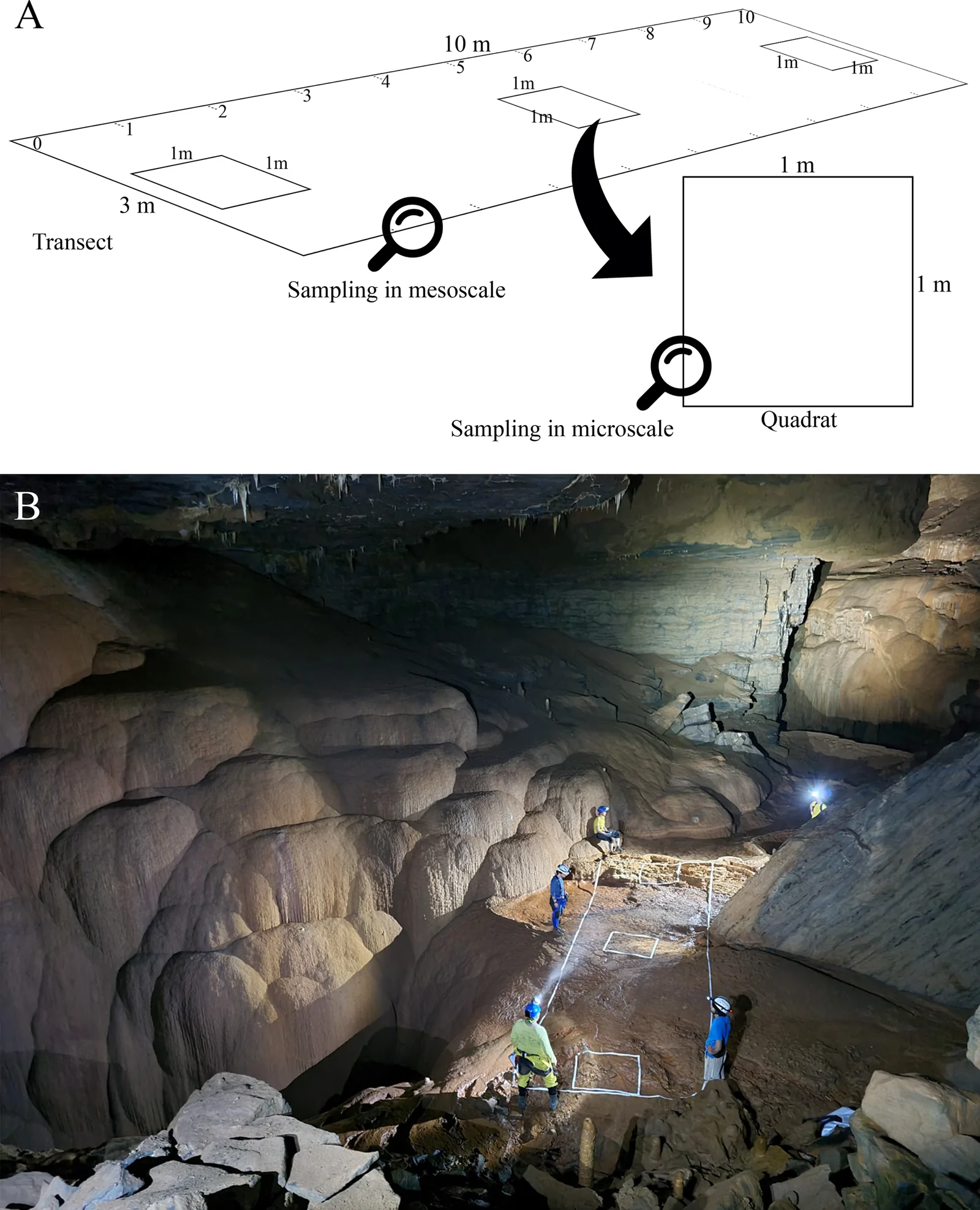
Sampling design. **A**–sampling design scheme showing mesoscale sampling (10 m x 30 m transect) and microscale sampling (1 m x 1 m quadrat); **B**–sampling design being applied in the ground of one of the caves

The composition (species identity and abundance) and richness of cave-dwelling invertebrates, together with habitat structural attributes, were assessed along 122 transects distributed across the floors of the 24 caves, extending from the entrance zones to deeper sectors (Figs. 1 and 2; Supplementary Material – Table S1). Transects represented a mesoscale sampling unit, each measuring 10 × 3 m. Within each transect, three quadrats (microscale sampling units; 1 m² each) were systematically placed, yielding a total of 366 quadrats (Fig. 2; Souza-Silva et al. 2021). This dual-scale approach was designed to capture distinct sets of environmental parameters that operate at different spatial extents. Given the small body size and limited mobility of the target invertebrates, the microscale captures the immediate microhabitat conditions, whereas the mesoscale encompasses broader environmental variations and resource availability that represent a significant spatial range for these organisms. Therefore, using both scales allows a more comprehensive assessment of habitat heterogeneity and its influence on community structure (Souza-Silva et al. 2021; Reis-Venâncio et al. 2024; Da Rocha Melo et al. 2025; Vaz et al. 2025).

Invertebrate sampling was conducted through active visual searches within both transects and quadrats by three collectors (Souza-Silva et al. 2021), while a fourth was dedicated exclusively to recording all abundances in situ. To minimize environmental impact, we collected only representative voucher specimens of each morphospecies per cave for laboratory identification, while the remaining individuals were just counted. Each researcher first sampled a specific quadrat and, immediately after, surveyed the adjacent transect area. This synchronized procedure ensured that each collector covered a distinct portion of the 30 m² transect, preventing sampling overlap and ensuring independent observations across both scales. Each sampling unit was considered complete only after a thorough inspection of the substrate surface and under all potential shelters (e.g., rocks, logs, and leaf litter). Because cave sectors differed markedly in structural complexity, the time devoted to searching each transect varied accordingly (Souza-Silva et al. 2021). In addition, targeted intuitive searches were conducted outside the standardized sampling units, prioritizing humid spots, organic resource accumulations, and potential shelters in order to improve the detection of troglobitic and stygobitic species (Souza-Silva et al. 2021). Specimens recorded outside standardized sampling units were excluded from all quantitative ecological analyses. Consequently, all stygobitic organisms were excluded from the statistical models.

Collected invertebrates were preserved in labeled vials containing 70% ethanol. In the laboratory, specimens were examined under a Stemi 508 stereomicroscope (ZEISS), identified to the lowest possible taxonomic level, and sorted into morphotypes following Oliver and Beattie (1996). To ensure taxonomic accuracy, specimen identification was performed using specific taxonomic keys, specialized monographs, and direct consultation with expert taxonomists (Supplementary Material – Table S2).

To ensure a rigorous classification of the fauna used in our models, potential troglobitic (terrestrial) and stygobitic (aquatic) species were identified based on the presence of morphological traits associated with prolonged evolutionary adaptation to subterranean environments (Culver and Pipan 2009; Souza-Silva et al. 2021; Zampaulo et al. 2025). Evaluated adaptations include reduction or loss of eyes and pigmentation, elongation of sensory and locomotor appendages, and modifications related to body size and sensory capacity (Sket 2008; Culver and Pipan 2009). We classified species as restricted to the subterranean based on the clear expression of troglomorphic traits, which were further evaluated and confirmed through extensive consultation with taxonomic specialists. For instance, although Hara and Pinto-Da-Rocha (2010) suggested that *Eusarcus cavernicola* may represent a complex of cryptic species that cannot be reliably distinguished based on external morphology alone, the population sampled from Gruta do Padre (and nearby related caves) was treated here as a distinct troglobitic lineage due to its marked morphological specialization.

To ensure accurate recognition of troglomorphic characteristics, taxonomic specialists in several groups (Araneae, Amblypygi, Amphipoda, Coleoptera, Collembola, Hemiptera, Isopoda, Orthoptera, Palpigradi, and Pseudoscorpiones) were consulted (see Acknowledgements and Supplementary Material – Table S2). Voucher specimens were deposited in the Collection of Subterranean Invertebrates of Lavras (ISLA), associated with the Center for Studies in Subterranean Biology (CEBS) at the Federal University of Lavras (UFLA).

While a comprehensive biological inventory was conducted for all encountered cave fauna (including opportunistic records of aquatic vertebrates like Osteichthyes), only invertebrate taxa were included in the subsequent ecological analyses.

### Environmental variables at different spatial scales

Habitat structural attributes along each transect were quantified following the protocol proposed by Souza-Silva et al. (2021). Air temperature and relative humidity were measured using a digital thermo-hygrometer placed on the ground at the center of each transect. To visually estimate the surface area covered by different organic and inorganic substrates, each transect was subdivided into ten contiguous sections measuring 1 × 3 m (Fig. 2). The total area occupied by each substrate type within a transect was obtained by summing the values recorded across all sections. To minimize observer bias, the same researcher characterized the substrates across all transects in all caves included in the study.

To quantify substrate composition at the microscale, high-resolution photographs (4000 × 3000 pixels) of each quadrat were taken at approximately chest height, using a Canon Powershot SX60HS camera positioned at a 90° angle relative to the ground surface (or as close as possible). These images were subsequently analyzed using ImageJ software (version 1.53k) to calculate the proportional area occupied by each substrate type within the quadrats.

Distances from transects and quadrats to the nearest cave entrance were obtained either through direct field measurements using a laser distance meter or, when available, by plotting transect locations on cave maps. Geographic distances among caves were calculated in QGIS 3.34 software using the Distance Matrix tool, based on the geographic coordinates of each cave.

### Data analysis

#### Environmental predictors

All substrates recorded within transects and quadrats were classified into predefined categories representing structural and biological components of the cave floor (detailed in Supplementary Material – Table S3). To capture distinct ecological dimensions of the subterranean habitat, we used these categories to calculate two types of metrics for each sampling unit: diversity and availability. Heterogeneity was calculated using the Shannon–Wiener index for: **Substrate Diversity**, which generally represents niche heterogeneity; **Shelter Diversity**, which reflects the architectural complexity of the microhabitat; and **Trophic Resources Diversity**, reflecting the variety of organic inputs. Additionally, we quantified Shelter Availability and Trophic Resources Availability by summing the total area (m²) of their respective substrate classes to measure the absolute volume of available niches. These metrics were evaluated alongside two primary physical and microclimatic predictors: **distance from the nearest cave entrance** and **microclimatic conditions** (air temperature and relative humidity), which directly constrain the physiological tolerances of subterranean fauna. Microclimatic variables were measured exclusively at the transect level and were omitted from microscale models.

### Variable screening and model implementation

All statistical analyses were conducted in RStudio (version 2023.06.1, Build 524). Transects with zero biological records were excluded from the statistical analyses, since ecological distance metrics, such as the Jaccard dissimilarity index, cannot be mathematically computed for pairs of sampling units containing exclusively zero values.

Prior to model construction, we examined collinearity among all initial predictors using the chart.Correlation function (PerformanceAnalytics 2.0.8 package) and Variance Inflation Factors (VIF; car 3.1-3 package), removing variables with **|r| > 0.70** or **VIF > 10**. When multicollinearity occurred, the selection between collinear variables was strictly guided by ecological relevance to cave biology, prioritizing stable microclimatic factors and direct resource availability over redundant spatial metrics. Crucially, we excluded a general **Substrate Diversity** metric from subsequent analyses because it was ecologically overbroad. Instead, using partitioned metrics such as shelter and trophic diversities better isolates the mechanism driving animal survival and resource partitioning in food-limited cave environments. Data normality was evaluated using the Shapiro–Wilk test. To assess the influence of geographic distance on community similarity, we performed a Mantel test comparing the faunal dissimilarity matrix with the geographical distance matrix between caves, via the vegan package. The final set of retained variables was assigned to subsequent models based on spatial scale. At the mesoscale (transects), the models included all screened physical features (**distance from the entrance**, **Shelter Diversity**, and **Shelter Availability**), trophic features (**Trophic Resources Diversity** and **Trophic Resources Availability**), and **microclimatic variables**. At the microscale (quadrats), the models included the same physical and trophic predictors, but microclimatic variables were excluded due to the absence of direct measurements.

To examine how these environmental predictors shape invertebrate communities, we fitted separate models for troglobitic and non-troglobitic assemblages across two complementary analyses. First, we employed a distance-based redundancy analysis (dbRDA; vegan 2.6-8 package) based on a Jaccard distance matrix (derived from presence-absence data) to evaluate shifts in species composition. Second, generalized linear mixed models (GLMMs) assuming a Poisson error distribution were used to evaluate effects on species richness. To test the global significance of each environmental predictor within the models, we performed Type-II Analysis of Variance (ANOVA) using Wald chi-square tests implemented via the car package. To account for spatial dependency, **Cave Identity** was included as a random factor for mesoscale models (transects), and **Transect** nested within **Cave** for microscale models (quadrats). Model overdispersion was checked via the performance package, and R² values were computed using the MuMIn 1.48.4 package. To ensure absolute statistical rigor and goodness-of-fit, we performed a comprehensive multi-metric model validation prior to parameter interpretation using the performance 0.17.0 package. We evaluated model residual homogeneity, presence of influential observations (outliers via leverage diagnostics), multicollinearity among environmental predictors using Variance Inflation Factors (VIF), and the probability distribution of quantile residuals. The complete diagnostic dashboards for all final models are provided in Supplementary Material – Fig. S1.

## Results

### Richness and composition of cave invertebrates

Across the 24 surveyed caves, including records from transects, quadrats, and additional targeted searches, a total of 2,754 specimens were recorded, representing 336 species distributed among 40 orders and at least 106 families. Araneae was the most species-rich and abundant group, comprising 67 species in 17 families and totaling 1,062 individuals. This was followed by Diptera and Coleoptera, with 49 and 37 species, respectively (Supplementary Material – Fig. S2, Table S3).

A total of 298 non-troglobitic species were recorded, distributed across 92 families. The assemblage was highly diverse, with a baseline composition dominated by a few hyper-diverse taxonomic groups. Araneae was the richest order, accounting for 63 species, followed closely by Diptera with 49 species, and Coleoptera with 35 species. Other representative insect orders included Hymenoptera (26 species), Hemiptera (15 species), Psocoptera (11 species), Lepidoptera (10 species), and Orthoptera (10 species).

### Cave-restricted species

**Fig. 3:**
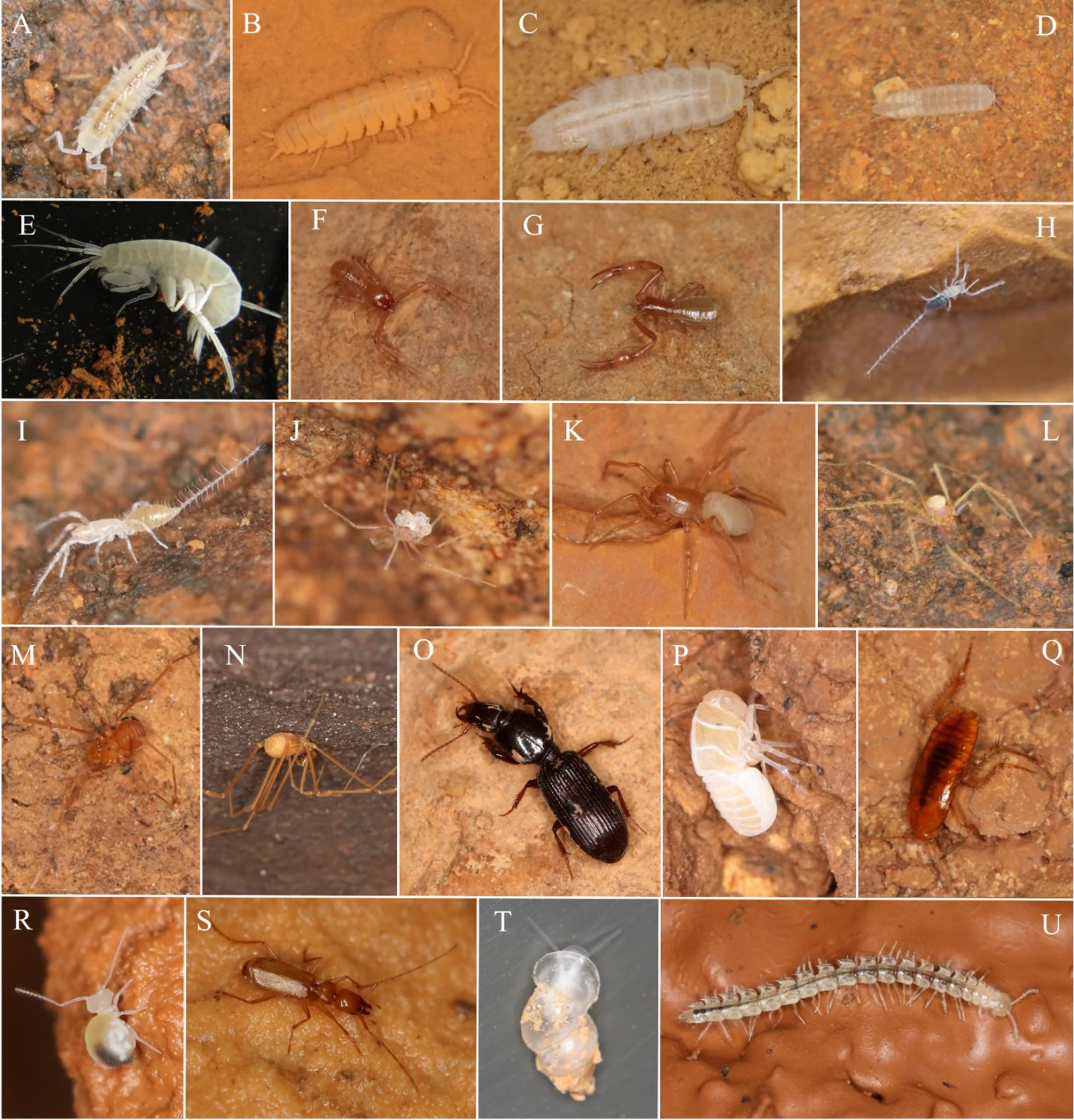
21 of the 36 troglobitic species found in the Santana region. **A**–*Trichorhina* sp.; **B**–*Chaimowiczia tatus*; **C**–*Xangoniscus paiabare*; **D**–Calabozoidea sp.1; **E**–*Spelaeogammarus santanensis*; **F**–*Pseudochthonius aware*; **G** – Ideoroncidae sp.1; **H** – *Eukoenenia* sp.1; **I** – *Eukoenenia* sp.2; **J** – Ochyroceratidae sp.1; **K** – Caponidae sp.1; **L** – Telemidae sp.1; **M** – Escadabiidae sp.1; **N** – *Eusarcus cf. cavernicola*; **O** – *Clivina* sp.1; **P** – Cixiidae sp.3; **Q** – Blattellidae sp.1; **R** – Arrhopalitidae sp.1; **S** – *Coarazuphium tessai*; **T** – *Potamolithus* sp.1; **U** – *Phaneromerium cavernicolum*

The surveyed caves harbored at least 38 obligate cave-dwelling species distributed across eight higher taxa and 18 families (Fig. 3; Table 1; Supplementary Material – Fig. S3, Table S3). These taxa comprised Crustacea (12 species), Arachnida (11 species), Hexapoda (9 species), Myriapoda (2 species), Mollusca (1 species), Nemertea (1 species), Annelida (1 species), and Osteichthyes (1 species) (Fig. 3; Table 1; Supplementary Material – Fig. S3, Table S3). Notably, 26 of the 38 troglobitic species were recorded in Padre Cave alone, accounting for nearly 70% of all cave-restricted species detected in the study area.

**Table 1:** Troglobitic species and morphotypes found in the 24 sampled caves, and the sampling scale where they were collected. *Ca* = Cave; *Qu* = Quadrat; *Sec* = Sector

Taxons	Species and Morphotypes	Ca	Qu	Sec
Amphipoda	*Spelaeogammarus santanensis*	+		
Araneae	Telemidae sp1	+		
	Ochyroceratidae sp1	+	+	+
	Ochyroceratidae sp2	+	+	
	Caponidae sp1	+		
Blattodea	Blattellidae sp1	+		
Coleoptera	Clivina sp1	+	+	+
	*Coarazuphium tessai*	+		
Copepoda	Copepoda sp1	+		
Entomobryomorpha	Paronellidae sp2	+	+	+
Gastropoda	*Potamolithus* sp1	+		
Hemiptera	Cixiidae sp3	+	+	+
Isopoda	*Chaimowiczia tatus*	+		
	Calabozoidea sp1		+	
	*Pectenoniscus santanensis*	+	+	+
	*Pectenoniscus* sp3			+
	Philosciidae	+		
	Platyarthridae sp1		+	+
	Styloniscidae sp1	+	+	+
	Styloniscidae sp2	+	+	+
	*Trichorhina* sp2			+
	*Xangoniscus paiabare*	+	+	+
Nemertea	Nemertea sp1	+		
Oligochaeta	Lumbricina sp3	+		
Opiliones	Escadabiidae sp1	+		+
	Escadabiidae sp2	+		
	*Eusarcus cf. cavernicola*	+	+	+
Orthoptera	Endecous sp1	+	+	+
Palpigradi	*Eukoenenia* sp1	+	+	+
	*Eukoenenia* sp2	+		
Poduromorpha	Poduromorpha sp1	+		
Pseudoscorpiones	Ideoroncidae sp1	+		+
	*Pseudochthonius aware*	+	+	+
Polydesmida	*Phaneromerium cavernicolum*	+	+	+
Symphyla	Symphyla sp1	+		
Symphypleona	Arrhopalitidae sp1	+	+	
	Arrhopalitidae sp2	+		
Siluriformes	*Pimelodella* sp1	+		

### Invertebrate assemblage structure at the mesoscale

At the mesoscale, Mantel tests revealed that geographic distance significantly influenced community composition for both non-troglobitic fauna (*p* < 0.001) and troglobitic fauna (*p* < 0.001). This indicates a significant geographic structuring in assemblage composition at this broader spatial scale (Supplementary Material – Fig. S4).

For non-troglobitic communities, the dbRDA model explained 43% of the total variation in species composition (*F* = 2.23, *p* = 0.001). Ordination results indicated that community structure was primarily associated with *cave identity* (*F* = 2.24, *p* = 0.001), *distance from the nearest entrance* (*F* = 2.91, *p* = 0.001), *shelter availability* (*F* = 1.97, *p* = 0.001), and *humidity* (*F* = 1.51, *p* = 0.017). Variation partitioning showed that environmental variables uniquely accounted for 25.0% of the explained variation, whereas spatial factors alone contributed negligibly. The shared fraction between environmental and spatial components explained 5.2% of the variation (Table 2; Supplementary Material – Fig. S5).

**Table 2:** Results for Distance-based Redundancy Analysis (dbRDA) for troglobitic and non-troglobitic fauna in meso and microscale. (Av = Availability)

	df	AIC	F	*p*	Explanation (%)
Troglobitic					
**Mesoscale**					
Cave	13	237.05	2.6520	0.0010	43.71
Distance	1	237.24	7.0444	0.0010	
Umidity	1	233.69	1.9677	0.0610	
**Microscale**					
Cave	7	222.07	1.6867	0.0030	19.66
Distance	1	218.68	2.3830	0.0210	
*N-Troglobitic*					
**Mesoscale**					
Cave	23	387.71	2.2482	0.0010	43.66
Distance	1	387.74	2.9150	0.0010	
Shelter Av.	1	388.98	1.9708	0.0010	
Umidity	1	391.01	1.5157	0.0170	
**Microscale**					
Cave	23	918.11	2.9993	0.0010	28.92
Distance	1	918.05	1.7457	0.0110	
Shelter.Av.	1	937.08	1.6840	0.0100	

For troglobitic fauna, the dbRDA model explained 44% of the variation in species composition (*F* = 2.89, *p* = 0.001). Community structure was significantly related to *cave identity* (*F* = 2.65, *p* = 0.001) and *distance from the nearest entrance* (*F* = 7.04, *p* = 0.001). Variation partitioning indicated that environmental variables uniquely explained 20.0% of the variation, whereas spatial factors and shared fractions contributed negligibly (Table 2; Supplementary Material – Fig. S5).

According to the GLMM for non-troglobitic communities, *distance from the nearest entrance* (β = −0.467, χ² = 8.099, *p* = 0.004) and *temperature* (β = −0.262, χ² = 4.937, *p* = 0.026) had significant negative effects on species richness. Although shelter availability was significant in the likelihood ratio test (*p* = 0.041), it was not supported by the Wald test (*p* = 0.098) and was therefore not considered a robust predictor. Conversely, humidity was significant in the Wald test (*p* = 0.002) but not in the likelihood ratio test (*p* = 0.105) and was likewise not interpreted as strongly supported. Overall, the model explained a substantial proportion of variance (marginal R² = 0.67; conditional R² = 0.83; delta method) (Table 3).

**Table 3:** Results for the generalized linear mixed models (GLMM) for troglobitic and non-troglobitic fauna at meso and microscale. Diversity (Div), availability (Av), distance from the entrance (distance). Troglobitic (Troglo), standard deviation (SE). R2m percent of the variance explained by the fixed effects and R2c percent of the variance explained by the fixed and random effects

Troglo	Estimate	Std Error	zvalue	*P*(t)	χ²	*P*_Anova_	Non-Troglo	Estimate β	Std Error	zvalue	*P*(z)	χ²	*P*_Anova_
Mesoscale													
Intercept	-0.3637	0.2500	-1.4550	0.1458			Intercept	1.2317	0.1172	10.5060	< 2E-16		
Distance	-0.0086	0.1162	-0.0740	0.9411	0.0055	0.9411	Distance	-0.4675	0.0994	-4.7040	2.55E-06	8.0989	0.0044
Temperature	0.0012	0.1435	0.0080	0.9935	0.0001	0.9935	Temperature	-0.2622	0.0750	-3.4990	0.0005	4.9373	0.0263
Humidity	0.3671	0.1768	2.0770	0.0378	4.3126	0.0378	Humidity	-0.2147	0.0691	-3.1050	0.0019	2.6211	0.1055
Shelter Div.	-0.0146	0.0978	-0.1490	0.8816	0.0222	0.8816	Shelter Div.	0.0557	0.0568	0.9800	0.3272	2.6868	0.1012
Trophic Div.	-0.1131	0.1354	-0.8360	0.4034	0.6981	0.4034	Trophic Div.	0.0607	0.0440	1.3800	0.1674	0.1808	0.6707
Shelter Av.	-0.0696	0.1066	-0.6530	0.5140	0.4260	0.5140	Shelter Av.	-0.1124	0.0680	-1.6530	0.0984	4.1545	0.0415
Trophic Av.	-0.1151	0.1793	-0.6420	0.5210	0.4119	0.5210	Trophic Av.	0.0370	0.0430	0.8600	0.3896	0.2657	0.6062
AIC	BIC	logLik	Deviance	df.resid			AIC	BIC	logLik	Deviance	df.resid		
340.6	365.8	-161.3	322.6	113			572.6	597.8	-277.3	554.6	113		
Dispersion	R²m		R²c				Dispersion	R²m		R²c			
0.979	0.1		0.23				0.814	0.67		0.83			
*Troglo*	Estimate	Std Error	zvalue	P(t)	χ²	P_Anova_	*Non-Troglo*	Estimate	Std Error	zvalue	P(z)	χ²	P_Anova_
Microscale													
Intercept	-2.0424	0.2028	-10.0700	< 2E-16			Intercept	-0.3802	0.1046	-3.6360	0.0003		
Distance	0.5811	0.1209	4.8070	1.53E-06	23.1083	1.53E-06	Distance	-1.0564	0.1541	-6.8570	7.05E-12	47.0135	7.05E-12
Shelter.Div.	-0.2457	0.1774	-1.3840	0.1660	1.9167	0.1662	Shelter.Div.	0.1112	0.0705	1.5760	0.1150	2.4839	0.1159
Trophic.Div.	0.1691	0.1559	1.0840	0.2780	1.1755	0.2783	Trophic.Div.	-0.0124	0.0435	-0.2850	0.7759	0.0810	0.7759
Shelter.Av.	0.0270	0.1580	0.1710	0.8640	0.0292	0.8643	Shelter.Av.	0.0990	0.0739	1.3410	0.1799	1.7985	0.1799
Trophic.Av.	-0.3032	0.3200	-0.9470	0.3430	0.8974	0.3435	Trophic.Av.	0.1953	0.0571	3.4220	0.0006	11.7080	0.0006
AIC	BIC	logLik	Deviance	df.resid			AIC	BIC	logLik	Deviance	df.resid		
380.9	408.3	-183.5	366.9	359			934.7	962	-460.4	920.7	359		
Dispersion	R²m		R²c				Dispersion	R²m		R²c			
0.687	0.09		0.18				0.672	0.53		0.65			

For troglobitic fauna, only *humidity* exerted a significant positive effect on species richness (β = 0.367, χ² = 4.313, *p* = 0.038). This model explained a relatively small proportion of variance (marginal R² = 0.10; conditional R² = 0.23; delta method).

### Invertebrate assemblage structure at the microscale

At the microscale, Mantel tests revealed that geographic distance significantly influenced community composition for both non-troglobitic fauna (*p* < 0.001) and troglobitic fauna (*p* < 0.001). This indicates a significant geographic structuring in assemblage composition at this local spatial scale (Supplementary Material – Fig. S4).

The dbRDA model for non-troglobitic assemblages explained 29.0% of the variation (*F* = 2.89, *p* = 0.001). Species composition was significantly associated with *cave identity* (*F* = 2.99, *p* = 0.001), *distance from the nearest entrance* (*F* = 1.74, *p* = 0.011), and *shelter availability* (*F* = 1.68, *p* = 0.010). Environmental variables uniquely explained 20,0% of the variation, while spatial factors again showed no independent effect. The shared environmental–spatial fraction accounted for 1.9% of the explained variation (Table 2; Supplementary Material – Fig. S5).

For the troglobitic fauna the dbRDA model explained 19.0% of the total variation (*F* = 1.77, *p* = 0.002), with species composition associated with *cave identity* (*F* = 1.68, *p* = 0.003) and *distance from the nearest entrance* (*F* = 2.38, *p* = 0.021). Environmental variables uniquely accounted for 4.6% of the explained variation, while spatial factors alone again showed no effect. The shared environmental–spatial fraction explained 2.4% of the variation (Table 2; Supplementary Material – Fig. S5).

**Fig. 4:**
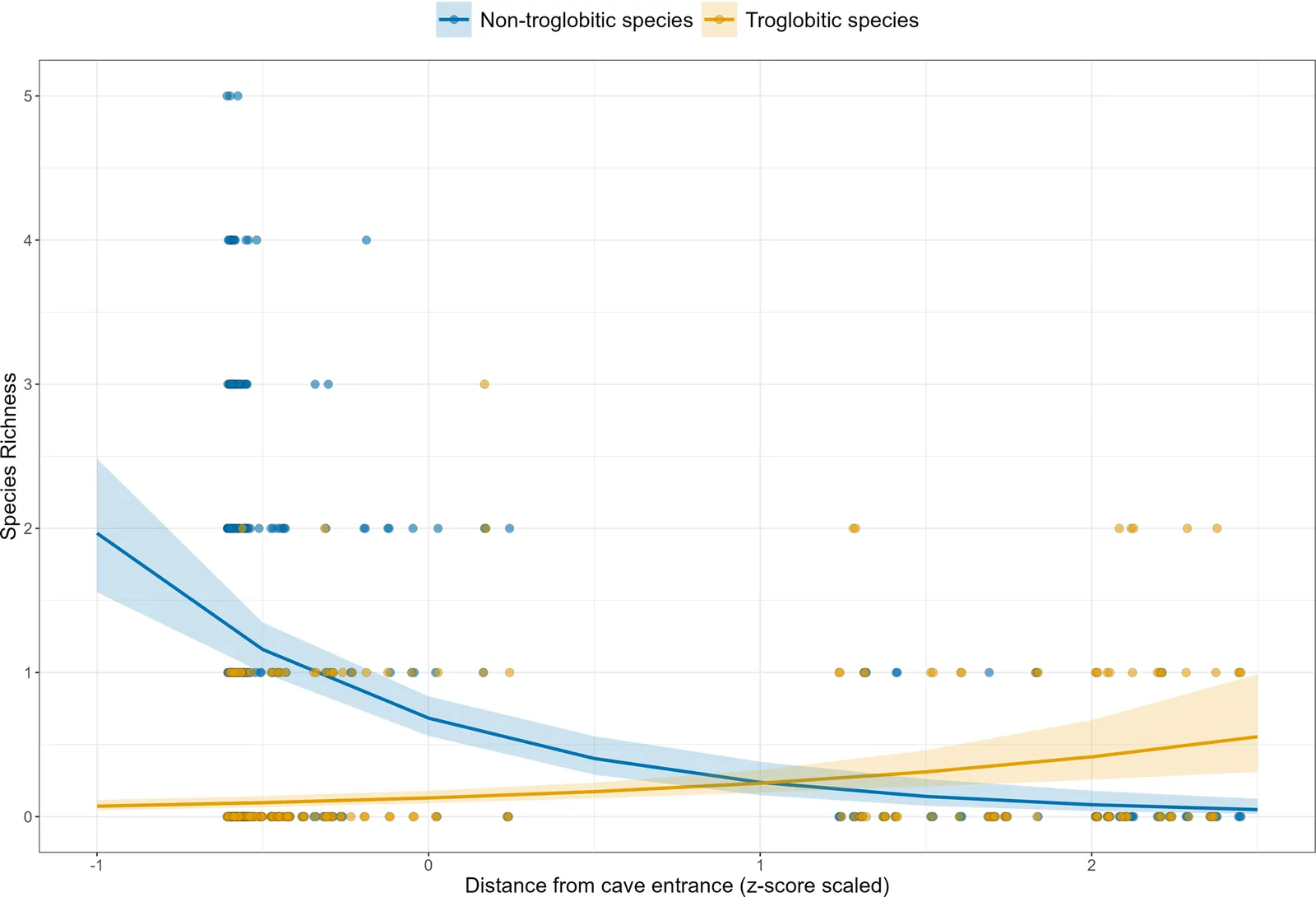
Effect of the distance from the nearest entrance on species richness for non-troglobitic (blue line) and troglobitic species (orange line). The distance is scaled to a z-score for better comparison. While the richness of non-troglobitic species decreases toward deeper areas of the cave, troglobitic species richness increases

Based on the GLMM, non-troglobitic richness was negatively affected by *distance from the nearest entrance* (β = −1.056, χ² = 47.013, *p* = 7.05 × 10⁻¹²) and positively associated with *trophic resource availability* (β = 0.195, χ² = 11.708, *p* = 6.22 × 10⁻⁴). This model also explained a substantial proportion of variance (marginal R² = 0.53; conditional R² = 0.65; delta method) (Fig. 4; Table 3).

Troglobitic richness was positively related solely to *distance from the nearest entrance* (β = 0.581, χ² = 23.108, *p* = 1.53 × 10⁻⁶), with the model explaining 9.0% of the variance (marginal R² = 0.09; conditional R² = 0.18; delta method) (Fig. 4; Table 3).

## Discussion

Our findings demonstrate that subterranean communities in the semi-arid Santana region are governed by a strict hierarchy of localized environmental filters, rather than regional spatial processes. While geographic distance between systems showed a negligible footprint in our variation partitioning models, community structure was heavily determined by cave identity and fine-scale internal gradients. This strong environmental filtering at meso and microscales contradicts the idea of subterranean community homogenization, positioning each cave as an ecologically singular unit (Mammola 2019; Bento et al. 2021).

### Scale-dependent responses to cave entrance gradients

Distance from the cave entrance consistently emerged as a dominant factor structuring subterranean communities across spatial scales, yet its ecological footprint was highly scale-dependent. For non-troglobitic communities, the negative effect of entrance distance on species richness intensifies from the mesoscale to the microscale. This indicates that while entrance proximity dictates general sector colonization by facultative fauna, due to broader mesoscale microclimatic fluctuations in temperature and light, it acts as an energy boundary at the fine scale (Prous et al. 2004; Souza-Silva et al. 2021). Deeper zones limit non-troglobitic richness because organic inputs originating from the epigean surface fade rapidly as distance from entrance increases (Tobin et al. 2013; Ficetola et al. 2018; Furtado et al. 2022). Altogether, the environmental gradients and high habitat heterogeneity typical of entrance zones progressively change to more homogeneous and simplified substrate conditions in deeper sectors, compounding this negative effect at the microscale (Prous et al. 2015; Souza-Silva et al. 2021).

Conversely, obligate troglobitic fauna displayed an inverse spatial sorting across scales. At the mesoscale, troglobitic richness was exclusively constrained by humidity, confirming that moisture acts as a coarse filter determining which zones can support specialized lineages characterized by thin cuticles and high susceptibility to desiccation (Lunghi et al. 2014; Kozel et al. 2019; Souza-Silva et al. 2021). Once inside a viable sector, however, moisture variance ceases to predict richness, and instead, troglobitic diversity becomes solely and positively driven by distance from the entrance at the microscale. Rather than sorting randomly on the cave floor, troglobites strictly track the deepest, most buffered microhabitats. This microscale restriction likely minimizes competitive interactions with energetically demanding non-troglobitic species, which fail to maintain high richness in these deep, oligotrophic areas (Sket 1999; Deharveng and Bedos 2000). While low resource availability in these deep zones constrains non-troglobitic fauna, the low energetic demands and K-selected life-history strategies of troglobitic species allow them to persist under prolonged food scarcity (Hüppop 2005; Souza-Silva et al. 2021).

### Environmental filtering overrides regional spatial structure

The constant significance of cave identity in our dbRDA models, explaining the unique compositional variation across both scales, challenges the assumption of regional community homogenization. Although Mantel tests detected significant spatial structuring in assemblage composition, our variation partitioning isolates purely spatial factors as negligible drivers of community composition. This clear asymmetry indicates that while regional subterranean connectivity through karst fissures and conduits may allow some regional dispersal (Rabelo et al. 2020; Pacheco et al. 2025), its capacity to homogenize communities across the landscape is effectively overridden by localized habitat filters (Mammola et al. 2016).

Consequently, geographic proximity does not appear to predict assemblage similarity in these semi-arid systems. Neighboring caves do not share identical communities because each system functions as a semi-independent ecological unit shaped by its own distinct matrix of geomorphology, microclimate, and food availability (Christman et al. 2005; Culver and Pipan 2009; Bento et al. 2021). These unique local attributes effectively override the effects of regional geographic distance, maintaining high species turnover (beta diversity) even at fine spatial scales (Mammola 2019; Veiga et al. 2025).

### Community responses to multiple habitat components and floor heterogeneity

Our dual-scale framework provided a critical analytical tool for understanding how physical structure and energy inputs operate across spatial dimensions. For non-troglobitic fauna, trophic resource availability emerged as a powerful positive predictor of species richness exclusively at the microscale, while failing to reach significance at the mesoscale. This statistical divergence exposes the highly patchy nature of energy subsidies, such as bat guano and plant debris, in semi-arid limestone caves (Ferreira and Martins 1999; Souza-Silva et al. 2011).

In permanently dry systems where alternative organic inputs are scarce, bat guano constitutes a major trophic subsidy that sustains entire invertebrate communities, triggering complex interactions and intricate food webs (Ferreira and Martins 1999; Ferreira 2019). However, guano deposition relies on bat populations that fluctuate seasonally in response to surface vegetation dynamics (Souza-Silva et al. 2011). Similarly, vegetal debris accumulates primarily during the dry season due to the deciduous vegetation of the Caatinga landscape, and is subsequently transported into the caves by rainy season flood pulses (Minshall et al. 1983; Crowther 1987; Brina 1998; Downes and Street 2005). Because of these surface-driven seasonal dynamics, trophic resources do not pool uniformly across cave sectors, and instead, they form localized energy patches on the cave floor. Thus, non-troglobitic organisms actively track and aggregate around these small spots (Schneider et al. 2011; Ladle et al. 2012; Pacheco et al. 2020 a).

Physical substrate heterogeneity, on the other hand, operated as a broader habitat filter. In our mesoscale dbRDA models, shelter availability significantly shaped community composition for non-troglobitic fauna, a trend that persisted into the microscale. Accumulations of diverse loose substrates create structural complexity and a mosaic of microhabitats that expand niche availability and micro-refuges, reducing competitive interactions and promoting niche partitioning among species (Prous et al. 2004; Souza-Silva et al. 2021). Troglobitic species, however, bypassed this reliance on structural and trophic patchiness. Their high specialization locks their distribution strictly to deep microclimatic stability, allowing them to avoid high-energy resource accumulations and consequent competitive displacement by energetically demanding non-troglobitic competitors (Sket 1999; Pacheco et al. 2020 a).

Finally, certain methodological boundaries regarding our sampling design should be acknowledged. Our standardized framework was restricted to cave floors, meaning that vertical substrates, such as high walls and ceilings, were not systematically surveyed, which could potentially underestimate specific groups that preferentially exploit upper cave zones. However, this limitation was actively mitigated by our additional, targeted intuitive searches outside the standardized units, which included potential hidden shelters, wall and low-ceiling microhabitats to maximize species detection. Therefore, while our standardized floor sampling represents a conservative snapshot of the community, we are confident that these results robustly capture the primary ecological filters operating in these systems. Nonetheless, our findings underscore that future studies integrating multi-layer sampling designs are highly critical to fully unraveling the spatial multidimensionality of subterranean communities in semi-arid karst landscapes.

### Conservation implications for subterranean biodiversity

The high community uniqueness and species turnover documented at transect and microhabitat scales have profound implications for environmental licensing and speleological legislation in Brazil (Pacheco et al. 2025; Zampaulo et al. 2025). Much of the original forest cover surrounding the caves in the Santana region has been replaced by pastures and monocultures. This surface deforestation directly diminishes the transport of plant debris into the cavities and indirectly reduces bat guano production by depleting external foraging resources, thereby threatening the persistence of entire subterranean food webs (Crowther 1987; Ferreira and Martins 1999; Cardoso et al. 2022).

Because our data reveal that high species turnover and community uniqueness are driven by internal cave heterogeneity rather than geographic distance, conservation strategies cannot rely on a regional paradigm, where protecting one cave is used to offset the environmental degradation of neighboring cavities. Since spatial proximity does not guarantee community similarity, individual caves cannot be treated as ecological substitutes for one another.

Under Brazilian the Brazilian speleological legislation (CECAV 2022), where cave protection levels are determined by categories of relevance, our scale-specific evidence shows that fine-scale inventories are non-negotiable. The discovery of highly specialized, isolated populations highlights that localized microhabitats harbor unique evolutionary trajectories (Hara & Pinto-Da-Rocha 2010; Junta et al. 2025). Ensuring that environmental impacts are assessed based on these localized scales is essential to prevent the irreversible loss of endemic and highly vulnerable subterranean biodiversity.

## Supplementary Information

Below is the link to the electronic supplementary material.

Supplementary Material 1

## Data Availability

The data supporting the findings of this study are available at https://github.com/vitorjunta/AreThereChoicesInTheDarkness. Additional data are available from the corresponding author upon reasonable request.

## Code availability

The R scripts used for data analyses are available at https://github.com/vitorjunta/AreThereChoicesInTheDarkness.
