# Supplementary material for: Are there choices in the darkness? habitat selection and environmental filtering shape invertebrate communities in semi-arid caves
Source: Oecologia. 2026 Jul 30;208(8):102. doi: 10.1007/s00442-026-05940-3 (PMC13424313; doi:10.1007/s00442-026-05940-3)
Supplement: Supplementary file 1 — Supplementary Material 1 [file 442_2026_5940_MOESM1_ESM.docx]

**Are there choices in the darkness? Habitat selection and environmental filtering shape invertebrate communities in semi-arid caves**

Vitor Gabriel Pereira Junta, Marconi Souza Silva, Rodrigo Lopes Ferreira

**Supplementary Material**


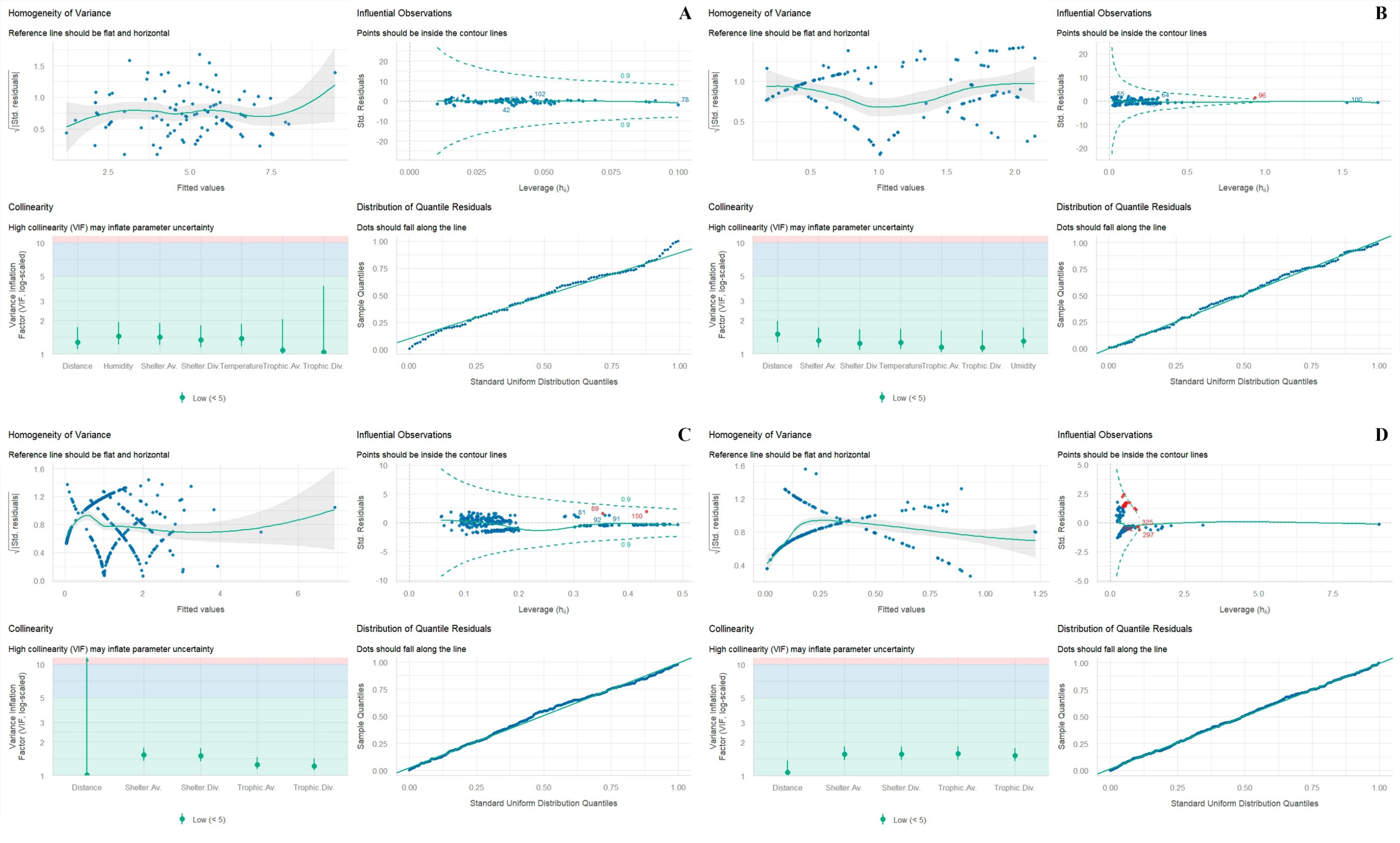


**Figure S1**: Multi-metric structural validation dashboards generated via the performance package for the four generalized linear mixed models (GLMMs) predicting species richness. (A) Non-troglobitic at the mesoscale (transects); (B) Troglobitic at the mesoscale (transects); (C) Non-troglobitic at the microscale (quadrats); and (D) Troglobitic at the microscale (quadrats). Each multi-plot dashboard independently assesses residual homogeneity (top-left), influential observations/leverage metrics (top-right), multicollinearity via Variance Inflation Factors (bottom-left), and quantile residual probability distributions (bottom-right). All diagnostic indicators confirm structural stability and compliance with statistical assumptions across scales. Shapiro–Wilk tests indicated that species richness was not normally distributed for either non-troglobitic (mesoscale: W = 0.944, p = 2.3 × 10⁻¹¹; microscale: W = 0.786, p < 2.2 × 10⁻¹⁶) or troglobitic fauna (mesoscale: W = 0.807, p = 2.3 × 10⁻¹¹; microscale: W = 0.486, p < 2.2 × 10⁻¹⁶), supporting the use of generalized linear mixed models. No evidence of overdispersion was detected for any model, either for non-troglobitic (mesoscale: dispersion ratio = 0.81, p = 0.90; microscale: 0.67, p = 1) or troglobitic assemblages (mesoscale: 0.98, p = 0.55; microscale: 0.69, p = 1) (Table 3). All final models successfully passed structural validation, displaying constant residual variance, absence of problematic multi-collinearity, and no distortive outlier effects

**
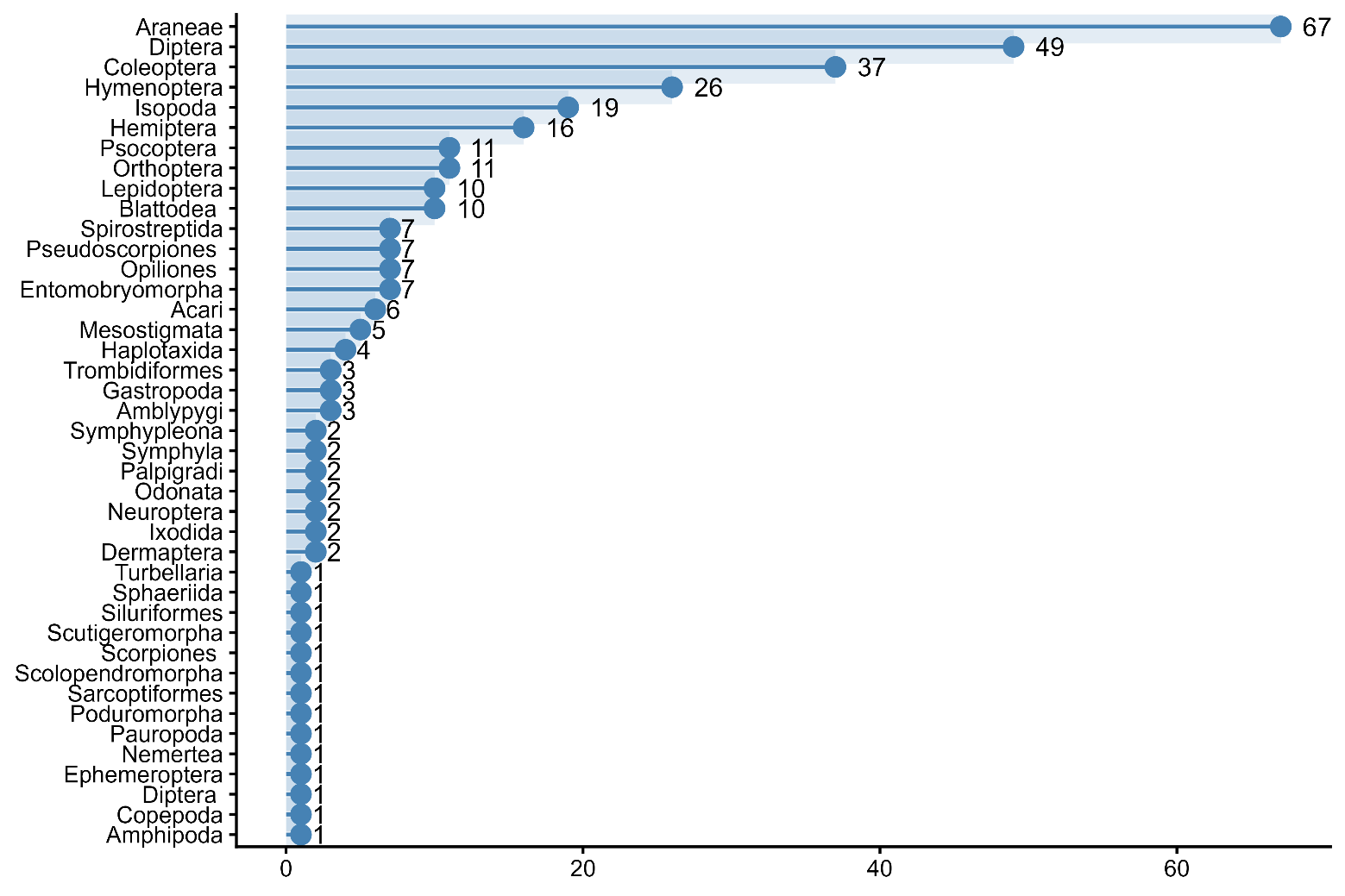
**

**Figure S2**: Species richness of the taxa found in the Santana region sampled caves. Araneae is the richest group (67 spp.), followed by Diptera (49 spp.) and Coleoptera (37 spp.).


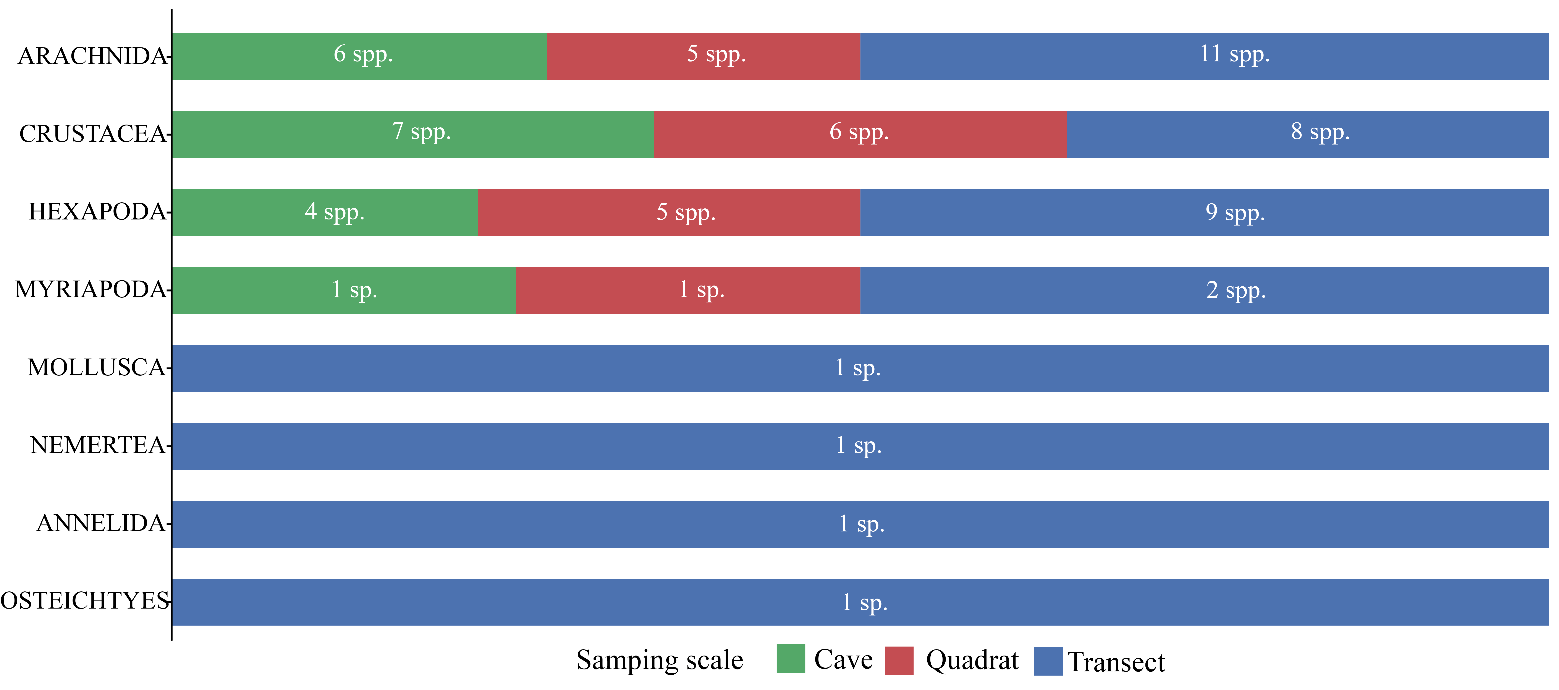


**Figure S3**: Richness of troglobitic species groups by sampling scale in the studied caves. Bars indicate the number of unique troglobitic species exclusively recorded or shared at each scale level: Cave (green), mesoscale (Transect; blue), and microscale (Quadrat; red).


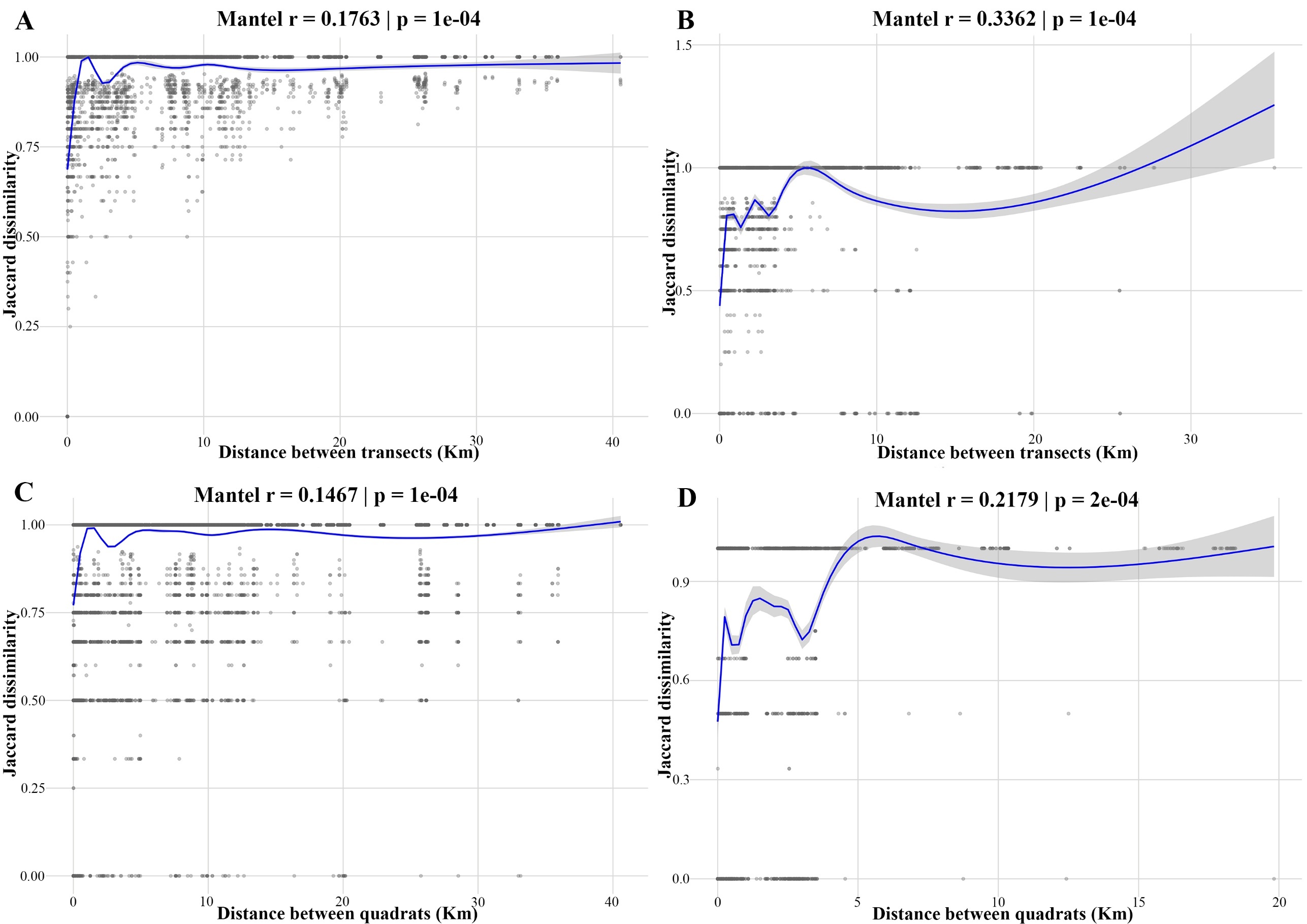


**Figure S4**: Distance-decay relationships (Jaccard dissimilarity vs. geographic distance) for subterranean invertebrate assemblages across sampling dimensions in the study area. (A) Non-troglobitic assemblages at the mesoscale (transects); (B) Troglobitic assemblages at the mesoscale (transects); (C) Non-troglobitic assemblages at the microscale (quadrats); and (D) Troglobitic assemblages at the microscale (quadrats). Blue lines represent smoothed trend curves, and gray areas indicate 95% confidence intervals. Mantel **r** and **p-values** are annotated on top of each plot.


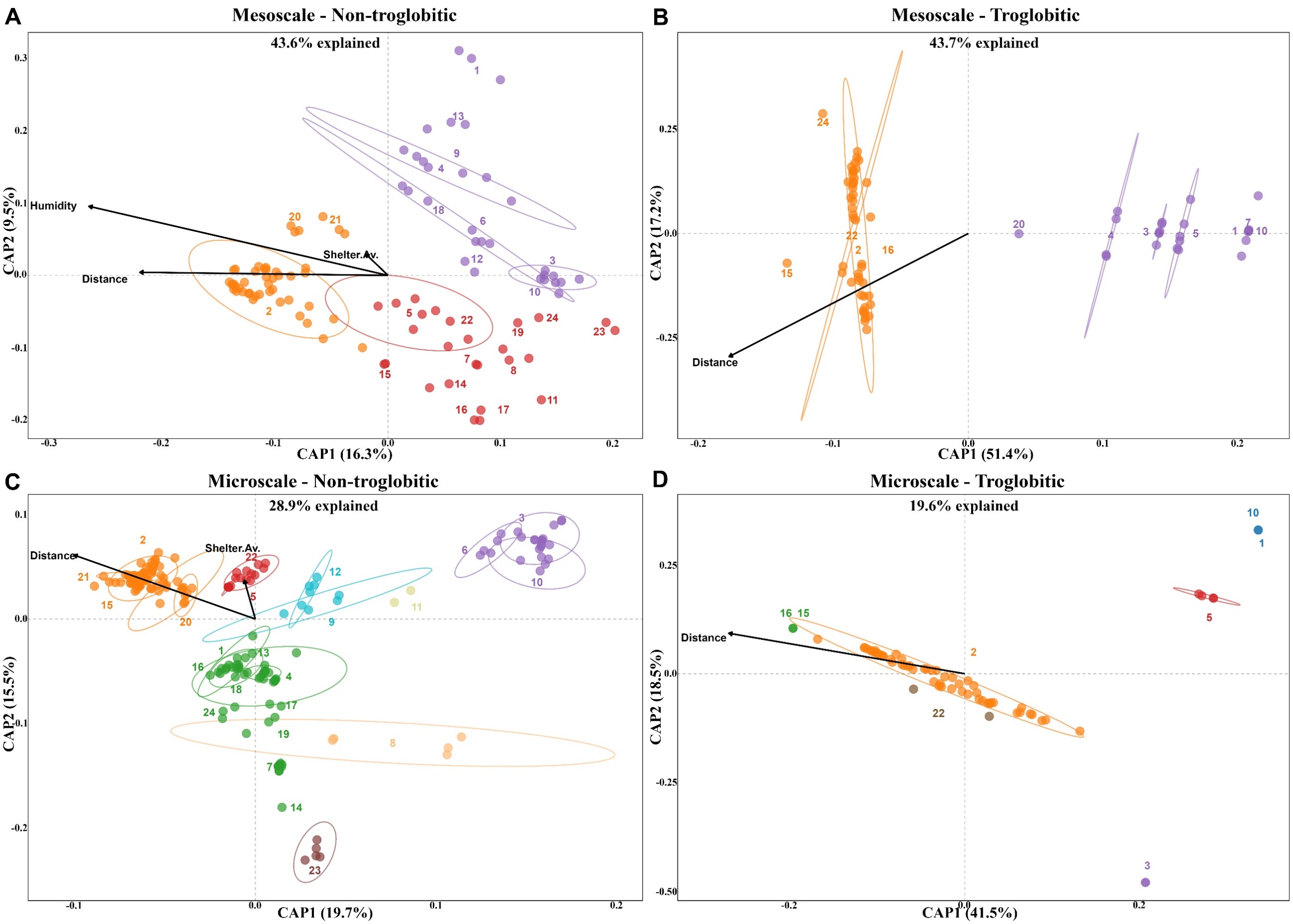


**Figure S5**: Distance-based Redundancy Analysis (dbRDA) ordinative plots based on Jaccard distance matrix, illustrating the effects of environmental predictors on non-troglobitic (A, C) and troglobitic (B, D) invertebrate assemblages across two spatial scales: mesoscale (transects; A, B) and microscale (quadrats; C, D). The percentage values displayed on the ordinations' axes (CAP1 and CAP2) represent the proportion of constrained (explained) variance accounted for by each axis, while the total percentage shown at the top of each panel indicates the cumulative variation explained by the complete set of explanatory variables in that specific model. Individual points represent sampling units (transects or quadrats), with numeric labels (1–24) indicating the respective cave identity. Colors and ellipses define clusters of similarity among sampling units calculated based on species composition. Black vectors (arrows) represent significant environmental predictors; the direction of the vector indicates the axis of maximum change for that variable, and its length is proportional to its explanatory power in structuring the community. Cave Index: 1 – Cânion da Baixa Verde Cave; 2 – Padre Cave; 3 – Labironto do Toxodon Cave; 4 – Boqueirão Cave; 5 – Pedra Escrevida Cave; 6 – Duas Cobras Cave; 7 – Tunel II Cave; 8 – São Geraldo Cave; 9 – Olho D’água do Cumbra Cave; 10 – Racha Bovina Cave; 11 – Tunel I Cave; 12 – Couve-Flor Cave; 13 – Geraldo Cruz Cave; 14 – Fenda Oblíqua Cave; 15 – Cedro Cave; 16 – Cedrão Cave; 17 – Cedrículo Cave; 18 – Pajeú Cave; 19 – Cristal Cave; 20 – Salobro Cave; 21 – Grota Cave; 22 – Leão Cave; 23 – Cinquentona Cave; 24 – Pedra Escrevidinha Cave.

**Table S1**: Comprehensive metadata of the studied caves and sampling units. **Cave ID**; **Cave** name; **Cave Long/Lat**: geographic coordinates in UTM 23S; **Basic Land Use**: dominant vegetation or anthropogenic matrix surrounding the cave entrance; **N. Transects**: total number of transects per cave; **Transects**: specific identification code for each sampling unit; and **Invertebrate** **Abundance**: total number of individuals; and **Invertebrate Species** **Richness**: total number of species recorded for each specific transect.

| **Cave ID** | **Cave** | **Cave Long** | **Cave Lat** | **Basic Land Use** | **N. Transects** | **Transects** | **Invertebrate Abundance** | **Invertebrate Species Richness** |
| --- | --- | --- | --- | --- | --- | --- | --- | --- |
| 1 | Cânion da Baixa Verde Cave | 596844 | 8537362 | Seasonal Dry Forest/Pasture Adjacent | 3 | S01 | 18 | 10 |
|  |  |  |  |  |  | S02 | 31 | 9 |
|  |  |  |  |  |  | S03 | 27 | 11 |
| 2 | Padre Cave | 601311 | 8538762 | Pasture/Seasonal Dry Forest | 53 | S01 | 27 | 7 |
|  |  |  |  |  |  | S02 | 11 | 6 |
|  |  |  |  |  |  | S03 | 36 | 6 |
|  |  |  |  |  |  | S04 | 13 | 6 |
|  |  |  |  |  |  | S05 | 2 | 2 |
|  |  |  |  |  |  | S06 | 2 | 2 |
|  |  |  |  |  |  | S07 | 12 | 6 |
|  |  |  |  |  |  | S08 | 10 | 4 |
|  |  |  |  |  |  | S09 | 2 | 2 |
|  |  |  |  |  |  | S10 | 24 | 7 |
|  |  |  |  |  |  | S11 | 9 | 2 |
|  |  |  |  |  |  | S12 | 23 | 7 |
|  |  |  |  |  |  | S13 | 370 | 14 |
|  |  |  |  |  |  | S14 | 15 | 5 |
|  |  |  |  |  |  | S15 | 7 | 5 |
|  |  |  |  |  |  | S16 | 24 | 6 |
|  |  |  |  |  |  | S17 | 155 | 8 |
|  |  |  |  |  |  | S18 | 81 | 7 |
|  |  |  |  |  |  | S19 | 27 | 9 |
|  |  |  |  |  |  | S20 | 73 | 12 |
|  |  |  |  |  |  | S21 | 80 | 11 |
|  |  |  |  |  |  | S22 | 11 | 6 |
|  |  |  |  |  |  | S23 | 11 | 5 |
|  |  |  |  |  |  | S24 | 3 | 2 |
|  |  |  |  |  |  | S25 | 21 | 6 |
|  |  |  |  |  |  | S26 | 4 | 4 |
|  |  |  |  |  |  | S27 | 0 | 0 |
|  |  |  |  |  |  | S28 | 0 | 0 |
|  |  |  |  |  |  | S29 | 30 | 3 |
|  |  |  |  |  |  | S30 | 28 | 6 |
|  |  |  |  |  |  | S31 | 34 | 8 |
|  |  |  |  |  |  | S32 | 11 | 4 |
|  |  |  |  |  |  | S33 | 10 | 2 |
|  |  |  |  |  |  | S34 | 2 | 2 |
|  |  |  |  |  |  | S35 | 0 | 0 |
|  |  |  |  |  |  | S36 | 3 | 3 |
|  |  |  |  |  |  | S37 | 0 | 0 |
|  |  |  |  |  |  | S38 | 0 | 0 |
|  |  |  |  |  |  | S39 | 0 | 0 |
|  |  |  |  |  |  | S40 | 0 | 0 |
|  |  |  |  |  |  | S41 | 0 | 0 |
|  |  |  |  |  |  | S42 | 30 | 8 |
|  |  |  |  |  |  | S43 | 48 | 11 |
|  |  |  |  |  |  | S44 | 77 | 5 |
|  |  |  |  |  |  | S45 | 41 | 9 |
|  |  |  |  |  |  | S46 | 11 | 5 |
|  |  |  |  |  |  | S47 | 4 | 4 |
|  |  |  |  |  |  | S48 | 12 | 5 |
|  |  |  |  |  |  | S49 | 18 | 7 |
|  |  |  |  |  |  | S50 | 3 | 2 |
|  |  |  |  |  |  | S51 | 5 | 2 |
|  |  |  |  |  |  | S52 | 19 | 7 |
|  |  |  |  |  |  | S53 | 19 | 4 |
| 3 | Labirinto do Toxodon Cave | 608884 | 8538822 | Seasonal Dry Forest/Pasture Adjacent | 7 | S01 | 33 | 13 |
|  |  |  |  |  |  | S02 | 11 | 7 |
|  |  |  |  |  |  | S03 | 12 | 8 |
|  |  |  |  |  |  | S04 | 6 | 5 |
|  |  |  |  |  |  | S05 | 7 | 6 |
|  |  |  |  |  |  | S06 | 8 | 5 |
|  |  |  |  |  |  | S07 | 12 | 7 |
| 4 | Boqueirão Cave | 597800 | 8537451 | Seasonal Dry Forest/Pasture Adjacent | 7 | S01 | 13 | 4 |
|  |  |  |  |  |  | S02 | 14 | 6 |
|  |  |  |  |  |  | S03 | 8 | 4 |
|  |  |  |  |  |  | S04 | 14 | 7 |
|  |  |  |  |  |  | S05 | 1 | 1 |
|  |  |  |  |  |  | S06 | 8 | 7 |
|  |  |  |  |  |  | S07 | 6 | 5 |
| 5 | Pedra Escrevida I Cave | 612496 | 8532062 | Seasonal Dry Forest | 8 | S01 | 36 | 4 |
|  |  |  |  |  |  | S02 | 45 | 5 |
|  |  |  |  |  |  | S03 | 1 | 1 |
|  |  |  |  |  |  | S04 | 4 | 4 |
|  |  |  |  |  |  | S05 | 2 | 2 |
|  |  |  |  |  |  | S06 | 2 | 2 |
|  |  |  |  |  |  | S07 | 8 | 4 |
|  |  |  |  |  |  | S08 | 0 | 0 |
| 6 | Duas Cobras Cave | 608351 | 8537716 | Seasonal Dry Forest | 2 | S01 | 10 | 8 |
|  |  |  |  |  |  | S02 | 30 | 11 |
| 7 | Tunel II Cave | 609390 | 8535922 | Seasonal Dry Forest | 3 | S01 | 68 | 8 |
|  |  |  |  |  |  | S02 | 10 | 4 |
|  |  |  |  |  |  | S03 | 17 | 2 |
| 8 | São Geraldo Cave | 609156 | 8535758 | Seasonal Dry Forest | 3 | S01 | 48 | 10 |
|  |  |  |  |  |  | S02 | 42 | 7 |
|  |  |  |  |  |  | S03 | 2 | 2 |
| 9 | Olho D'água do Cumbra Cave | 601050 | 8529525 | Seasonal Dry Forest/Pasture Adjacen | 2 | S01 | 20 | 8 |
|  |  |  |  |  |  | S02 | 19 | 9 |
| 10 | Racha Bovina Cave | 615975 | 8531872 | Seasonal Dry Forest/Pasture Adjacen | 2 | S01 | 31 | 14 |
|  |  |  |  |  |  | S02 | 19 | 5 |
| 11 | Tunel I Cave | 609348 | 8535882 | Seasonal Dry Forest | 1 | S01 | 9 | 5 |
| 12 | Couve-Flor Cave | 609111 | 8535870 | Seasonal Dry Forest | 3 | S01 | 2 | 2 |
|  |  |  |  |  |  | S02 | 1 | 1 |
|  |  |  |  |  |  | S03 | 0 | 0 |
| 13 | Geraldo Cruz Cave | 600656 | 8528434 | Seasonal Dry Forest | 2 | S01 | 11 | 5 |
|  |  |  |  |  |  | S02 | 7 | 4 |
| 14 | Fenda Oblíqua Cave | 600891 | 8528568 | Seasonal Dry Forest/Pasture Adjacen | 1 | S01 | 3 | 3 |
| 15 | Cedro Cave | 601082 | 8538937 | Seasonal Dry Forest | 2 | S01 | 35 | 7 |
|  |  |  |  |  |  | S02 | 10 | 7 |
| 16 | Cedrão Cave | 601040 | 8539058 | Seasonal Dry Forest | 3 | S01 | 61 | 12 |
|  |  |  |  |  |  | S02 | 57 | 7 |
|  |  |  |  |  |  | S03 | 21 | 6 |
| 17 | Cedrículo Cave | 601044 | 8539067 | Seasonal Dry Forest | 1 | S01 | 67 | 10 |
| 18 | Pajeú Cave | 598760 | 8526941 | Seasonal Dry Forest | 5 | S01 | 29 | 11 |
|  |  |  |  |  |  | S02 | 42 | 6 |
|  |  |  |  |  |  | S03 | 19 | 5 |
|  |  |  |  |  |  | S04 | 19 | 6 |
|  |  |  |  |  |  | S05 | 5 | 3 |
| 19 | Cristal Cave | 599497 | 8527612 | Seasonal Dry Forest | 1 | S01 | 53 | 9 |
| 20 | Salobro Cave | 588687 | 8554258 | Seasonal Dry Forest/Pasture Adjacent | 3 | S01 | 54 | 8 |
|  |  |  |  |  |  | S02 | 24 | 11 |
|  |  |  |  |  |  | S03 | 10 | 8 |
| 21 | Grota Cave | 587858 | 8561161 | Seasonal Dry Forest/Pasture Adjacent | 3 | S01 | 17 | 10 |
|  |  |  |  |  |  | S02 | 88 | 11 |
|  |  |  |  |  |  | S03 | 61 | 5 |
| 22 | Leão Cave | 601365 | 8539161 | Seasonal Dry Forest/Pasture Adjacent | 4 | S01 | 20 | 10 |
|  |  |  |  |  |  | S02 | 38 | 12 |
|  |  |  |  |  |  | S03 | 14 | 9 |
|  |  |  |  |  |  | S04 | 1 | 1 |
| 23 | Cinquentona Cave | 605268 | 8538657 | Seasonal Dry Forest | 2 | S01 | 30 | 5 |
|  |  |  |  |  |  | S02 | 65 | 7 |
| 24 | Pedra Escrevidinha Cave | 605264 | 8538648 | Seasonal Dry Forest | 1 | S01 | 109 | 16 |
|  |  |  |  |  |  |  |  |  |

**Table S2**: Taxonomic references, identification keys, and consulting specialists utilized for the morphological identification and morphospecies sorting of subterranean invertebrate taxa.

| **Taxon** | **Reference** | **Specialist** |
| --- | --- | --- |
| **Acari** | FLECHTMANN, Carlos HW. Ácaros de importância médico-veterinária. Biblioteca rural, 1973. | Leopoldo Ferreira de Oliveira Bernardi |
| **Amblypygi** | PINTO-DA-ROCHA, Ricardo; MACHADO, Glauco; WEYGOLDT, Peter. Two new species of Charinus Simon, 1892 from Brazil with biological notes (Arachnida; Amblypygi; Charinidae). Journal of Natural History, v. 36, n. 1, p. 107-118, 2002. | Richard Torres |
| **Amphipoda** | BASTOS-PEREIRA, Rafaela; FERREIRA, Rodrigo L. Spelaeogammarus uai (Bogidielloidea: Artesiidae): a new troglobitic amphipod from Brazil. Zootaxa, v. 4231, n. 1, p. 38-50, 2017. | Rafaela Bastos Pereira & Giovanna Monticelli Cardoso |
| **Araneae** | BRESCOVIT, Antonio Domingos et al. Araneae. Amazonian Arachnida and Myriapoda. Pensoft, Sofia, p. 303-344, 2002. | Vinicius Sérgio Rodrigues Diniz |
|  |  |  |
|  | JOCQUÉ, Rudy et al. Spider families of the world. 2006. |  |
|  |  |  |
|  | ROTH, Vincent D. A Guide to Spiders and Their Kin. 1969. |  |
| **Archaeognatha** | ALE-ROCHA, Rosaly; ADIS, Joachim. Archaeognatha Börner, 1904. In: RAFAEL, José Albertino; MELO, Gabriel Augusto Rodrigues; CARVALHO, Claudio José Barros de; CASARI, Sônia Aparecida; CONSTANTINO, Reginaldo (org.). Insetos do Brasil: diversidade e taxonomia. Ribeirão Preto: Holos Editora, 2012. p. 219–223. |  |
| **Blattodea** | GRANDCOLAS, P.; PELLENS, R. Baratas (Blattaria excluindo térmitas). In: RAFAEL, J. A. et al. (Eds.). Insetos do Brasil: diversidade e taxonomia. Ribeirão Preto: Holos Editora, 2012. p. 333–346. |  |
| **Coleoptera** | CASARI, S. A.; BIFFI, G.; IDE, S. Coleoptera. In: RAFAEL, J. A. et al. (Eds.). Insetos do Brasil: diversidade e taxonomia. Ribeirão Preto: Holos Editora, 2012. p. 454–535. | Thais Pellegrini |
|  |  |  |
|  | MARSHALL, S. A. Beetles: the natural history and diversity of Coleoptera. Firefly Books, 2018. |  |
| **Collembola** | DE SOUZA, Paolla Gabryelle Cavalcante et al. A highly troglomorphic new genus of Sminthuridae (Collembola, Symphypleona) from the Brazilian semiarid region. Insects, v. 13, n. 7, p. 650, 2022. | Paolla Gabryelle Cavalcante de Souza |
|  | SOUZA, Paolla Gabryelle Cavalcante de. Revisão de Trogolaphysa Mills,(Collembola, Entomobryomorpha, Paronellidae), com a descrição de novas espécies de cavernas, domínio caatinga, Brasil. 2021. |  |
|  |  |  |
|  | CIPOLA, N. G.; SILVA, D. D. da; BELLINI, B. C. Class Collembola. In: HAMADA, N.; THORP, J. H.; ROGERS, D. C. (Eds.). Thorp and Covich's Freshwater Invertebrates. 4. ed. Academic Press, 2018. cap. 2, p. 11–55. DOI https://doi.org/10.1016/B978-0-12-804223-6.00002-0. |  |
| **Dermaptera** | HAAS, F. Dermaptera. In: RAFAEL, J. A. et al. (Eds.). Insetos do Brasil: diversidade e taxonomia. Ribeirão Preto: Holos Editora, 2012. p. 297–305. |  |
| **Diplura** | MORAIS, José Wellington de; FIGUEIREDO, Annelyse Rosenthal; ADIS, Joachim. Diplura Börner, 1904. In: RAFAEL, José Albertino; MELO, Gabriel Augusto Rodrigues; CARVALHO, Claudio José Barros de; CASARI, Sônia Aparecida; CONSTANTINO, Reginaldo (org.). Insetos do Brasil: diversidade e taxonomia. Ribeirão Preto: Holos Editora, 2012. p. 213–218. |  |
| **Diptera** | CARVALHO, C. J. B. de et al. Diptera. In: RAFAEL, J. A. et al. (Eds.). Insetos do Brasil: diversidade e taxonomia. Ribeirão Preto: Holos Editora, 2012. p. 701–743. |  |
| **Formicidae** | BACCARO, F. B. et al. Guia para os gêneros de formigas do Brasil. Manaus: Editora INPA, 2015. 388 p. DOI doi.org. |  |
| **Gastropoda** | BOUCHET, Philippe et al. Classification and nomenclator of gastropod families. 2005. |  |
| **Hemiptera** | GRAZIA, J. et al. Hemiptera. In: RAFAEL, J. A. et al. (Eds.). Insetos do Brasil: diversidade e taxonomia. Ribeirão Preto: Holos Editora, 2012. p. 348–405. | Julio Cesar Vaz |
| **Hymenoptera** | MELO, G. A. R.; AGUIAR, A. P.; GARCETE-BARRETT, B. R. Hymenoptera. In: RAFAEL, J. A. et al. (Eds.). Insetos do Brasil: diversidade e taxonomia. Ribeirão Preto: Holos Editora, 2012. p. 553–612. |  |
| **Isopoda** | CARDOSO, Giovanna Monticelli et al. New cave species of Pectenoniscus Andersson, 1960 (Isopoda: Oniscidea: Styloniscidae) and an identification key for the genus. Nauplius, v. 28, p. e2020039, 2020. | Giovanna Monticelli Cardoso |
| **Isoptera** | CONSTANTINO, R. Isoptera. In: RAFAEL, J. A. et al. (Eds.). Insetos do Brasil. Ribeirão Preto: Holos Editora, 2012. p. 312–321. |  |
| **Immature Coleoptera** | ROBERTS, A. R. (1930). A key to the principal families of Coleoptera in the larval stage. Bulletin of Entomological Research, 21(1), 57-72. |  |
|  |  |  |
|  | ALMEIDA, L. M., & MISE, K. M. (2009). Diagnosis and key of the main families and species of South American Coleoptera of forensic importance. Revista Brasileira de Entomologia, 53(2), 227-244. |  |
| **Immature Diptera** | BORKENT, A., & ROTHERAY, G. (2009). Key to Diptera families—larvae. Manual of Central American Diptera, 1, 157-191. |  |
| **Immature Lepidoptera** | WILLIAMS, J. R. (1953). The larvae and pupae of some important Lepidoptera. Bulletin of Entomological Research, 43(4), 691-701. |  |
|  |  |  |
|  | THYSSEN, P. J. (2009). Keys for identification of immature insects. In Current concepts in forensic entomology (pp. 25-42). Dordrecht: Springer Netherlands. |  |
| **Immature Neuroptera** | HECKMAN, C. W. (2017). Neuroptera (including megaloptera). Springer. |  |
| **Lepidoptera** |  |  |
|  | DUARTE, M. et al. Lepidoptera. In: RAFAEL, J. A. et al. (Eds.). Insetos do Brasil: diversidade e taxonomia. Ribeirão Preto: Holos Editora, 2012. p. 625–681. |  |
| **Myriapoda** | TRAJANO, Eleonora et al. Synopsis of Brazilian cave-dwelling millipedes (Diplopoda). Papéis avulsos de Zoologia, v. 41, n. 1-28 (1999-2001), p. 259-287, 1999. |  |
|  |  |  |
|  | ENGHOFF, Henrik et al. Diplopoda—taxonomic overview. Treatise on Zoology-Anatomy, Taxonomy, Biology. The Myriapoda, Volume 2, p. 363-453, 2015. |  |
|  |  |  |
|  | ADIS, J. (Ed.). Amazonian Arachnida & Myriapoda: identification keys to all classes, orders, families, some genera and lists of known terrestrial species. Sofia: Pensoft Publishers, 2002. 590 p. (Pensoft Series Faunistica, n. 24). |  |
| **Neuroptera** | FREITAS, S. de; PENNY, N. D. Neuroptera. In: RAFAEL, J. A. et al. (Eds.). Insetos do Brasil: diversidade e taxonomia. Ribeirão Preto: Holos Editora, 2012. p. 538–546. |  |
| **Oligochaeta** | SIMS, R.W. (1980) A classifications and the distribution of earthworms, suborder Lumbricina (Haplotaxida: Oligochaeta). |  |
| **Opilionida** | KURY, A. B.; PINTO-DA-ROCHA, R. Opiliones. In: ADIS, J. (Ed.). Amazonian Arachnida & Myriapoda: identification keys to all classes, orders, families, some genera and lists of known terrestrial species. Sofia: Pensoft Publishers, 2002. p. 345–362. (Pensoft Series Faunistica, n. 24). |  |
|  |  |  |
|  | HARA, M. R.; PINTO-DA-ROCHA, R. Systematic review and cladistic analysis of the genus Eusarcus Perty 1833 (Arachnida, Opiliones, Gonyleptidae). Zootaxa, v. 2698, n. 1, p. 1–136, 6 dez. 2010. DOI doi.org. |  |
| **Orthoptera** | SPERBER, Carlos Frankl; MEWS, Carina Marciela; LHANO, Marcos Gonçalves; CHAMORRO, Juliana; MESA, Alejo. Orthoptera Olivier, 1791. In: RAFAEL, José Albertino; MELO, Gabriel Augusto Rodrigues; CARVALHO, Claudio José Barros de; CASARI, Sonia Antonia; CONSTANTINO, Reginaldo (org.). Insetos do Brasil: diversidade e taxonomia. Ribeirão Preto: Holos Editora, 2012. p. 271–287. | Vitor Gabriel Pereira Junta |
| **Palpigradi** | MAYORAL, Jaime G. Clase Arachnida: Orden Palpigradi. Revista electrónica IDE@ - SEA, Ibero Diversidad Entomológica @ccesible, Zaragoza, n. 10, p. 1–9, 30 jun. 2015. | Maysa Fernanda Villela Rezende Souza |
| **Protura** | RAFAEL, José Albertino; BEDANO, José Camilo. Protura Silvestri, 1907. In: RAFAEL, José Albertino; MELO, Gabriel Augusto Rodrigues; CARVALHO, Claudio José Barros de; CASARI, Sônia Aparecida; CONSTANTINO, Reginaldo (org.). Insetos do Brasil: diversidade e taxonomia. Ribeirão Preto: Holos Editora, 2012. p. 197–200. |  |
| **Pseudoescorpiones** | HARVEY, M.S. (1992) The phylogeny and classification of the Pseudoscorpionida (Chelicerata: Arachnida). Invertebrate | Guilherme Prado |
|  | Taxonomy, 6, 1373–1435. |  |
|  | CHAMBERLIN, J.C. (1931) The arachnid order Chelonethida. Stanford University Publications, (Biological Sciences) 7 (1), 1 |  |
|  | 284 |  |
| **Psocoptera** | GARCÍA ALDRETE, A. N.; MOCKFORD, E. L. Psocoptera. In: RAFAEL, J. A. et al. (Eds.). Insetos do Brasil: diversidade e taxonomia. Ribeirão Preto: Holos Editora, 2012. p. 423–437. |  |
|  |  |  |
|  | LIENHARD, Charles; FERREIRA, Rodrigo L. Review of Brazilian cave psocids of the families Psyllipsocidae and Prionoglarididae (Psocodea:‘Psocoptera’: Trogiomorpha) with a key to the South American species of these families. Revue suisse de Zoologie, v. 122, n. 1, p. 121-142, 2020. |  |
| **Scorpiones** | BÜCHERL, W. Escorpiões e escorpionismo no Brasil. X Catálogo da coleção escorpiônica |  |
|  | do Instituto Butantã. Memórias do Instituto Butantã. São Paulo, v 29, p. 255-275, 1959. |  |
|  |  |  |
|  | LOURENÇO, Wilson R. Description of a new species of Tityus (Scorpiones, Buthidae) from Serra da Jurema in the State of Bahia, Brazil. 2003. |  |
| **Siluriformes** | FERNÁNDEZ, L., & BICHUETTE, M. E. (2002). A new cave dwelling species of Ituglanis from the São Domingos karst, central Brazil (Siluriformes: Trichomycteridae). Ichthyological Exploration of Freshwaters, 13(3), 273-278. |  |
| **Turbellaria** | MARCUS, Ernesto. Turbellaria do Brasil. Boletim da Faculdade de Filosofia, Ciências e Letras da Universidade de São Paulo, Zoologia, São Paulo, v. 13, p. 111–207, 1948 |  |
| **Zygentoma** | HENRIQUES, Augusto L.; MENDES, Luis F. Zygentoma Börner, 1904. In: RAFAEL, José Albertino; MELO, Gabriel Augusto Rodrigues; CARVALHO, Claudio José Barros de; CASARI, Sônia Aparecida; CONSTANTINO, Reginaldo (org.). Insetos do Brasil: diversidade e taxonomia. Ribeirão Preto: Holos Editora, 2012. p. 225–230. |  |

**Table S3**: Substrate classification and metric assignment. Categories are explicitly designated based on their functional role for the cave fauna, defining whether they were used to calculate Trophic Resources (Diversity and Availability) or Shelter (Diversity and Availability) metrics in the final statistical models.

| **Acronym** | **Substrate** | **Metric Assignment** | **Acronym** | **Substrate** | **Metric Assignment** |
| --- | --- | --- | --- | --- | --- |
| GU | guano | Trophic Resources (Calculated & Modeled) | MB | medium rock (500 - 1000 mm) | Shelter (Calculated & Modeled) |
| FZ | feces | Trophic Resources (Calculated & Modeled) | SB | small rock (250 - 500mm) | Shelter (Calculated & Modeled) |
| CRC | carcass | Trophic Resources (Calculated & Modeled) | CB | cobbles (64 - 250mm) | Shelter (Calculated & Modeled) |
| RZ | roots | Trophic Resources (Calculated & Modeled) | CAG | coarse gravel (16 - 64mm) | Shelter (Calculated & Modeled) |
| SER | litter | Trophic Resources (Calculated & Modeled) | CAF | fine gravel (2 - 16mm) | Shelter (Calculated & Modeled) |
| DTV | vegetal debris (< 10 mm) | Trophic Resources (Calculated & Modeled) | ARE | sand (0.06 - 2mm) | General Substrate Only (Not included in final models) |
| GALF | fine branch (11 - 30 mm) | Trophic Resources (Calculated & Modeled) | SEF | silt (≤ 0.05 mm) | General Substrate Only (Not included in final models) |
| GALM | medium branch (31 - 50 mm) | Trophic Resources (Calculated & Modeled) | HP | hardpan | General Substrate Only (Not included in final models) |
| GALG | coarse branch (51 - 250 mm) | Trophic Resources (Calculated & Modeled) | ES | speleothems | General Substrate Only (Not included in final models) |
| TRO | trunk (> 250 mm) | Trophic Resources (Calculated & Modeled) | JNS | calcite rafts | General Substrate Only (Not included in final models) |
| TM | termite mounts | Shelter (Calculated & Modeled) | JNC | concreted calcite raft | General Substrate Only (Not included in final models) |
| ST | water streams | Shelter (Calculated & Modeled) | ESTC | stalactite | General Substrate Only (Not included in final models) |
| WP | water pond | Shelter (Calculated & Modeled) | EST | stalagmite | General Substrate Only (Not included in final models) |
| DP | drip water | Shelter (Calculated & Modeled) | MTR | micro travertine | Shelter (Calculated & Modeled) |
| FG | phanerogams | Trophic Resources (Calculated & Modeled) | TRA | travertine | Shelter (Calculated & Modeled) |
| ACT | actinomycetes | Trophic Resources (Calculated & Modeled) | ESR | rough flowstone | General Substrate Only (Not included in final models) |
| OTO | another organic substrate | General Substrate Only (Not included in final models) | ESC | flowstone | General Substrate Only (Not included in final models) |
| RC | concrete floor | General Substrate Only (Not included in final models) | BM | worm acorn | General Substrate Only (Not included in final models) |
| RR | rough rock | General Substrate Only (Not included in final models) | GRR | retraction cracks | Shelter (Calculated & Modeled) |
| XB | large rock (1000 - 4000 mm) | Shelter (Calculated & Modeled) | COG | gastropod shell | Shelter (Calculated & Modeled) |

**Table S4**: List of invertebrate morphospecies recorded across 24 caves in the study area. Taxonomic identification, habitat type, troglomorphic status, and occurrence are provided. **Quad:** Total abundance recorded within 1 m² quadrats (microscale); **Tran:** Total abundance recorded along 3m x 10m transects (mesoscale); **Add:** Additional records obtained through searching outside standardized sampling units. **Troglobitic species** are highlighted in bold. **Occurrence**: 1 – Cânion da Baixa Verde Cave; 2 – Padre Cave; 3 – Labironto do Toxodon Cave; 4 – Boqueirão Cave; 5 – Pedra Escrevida Cave; 6 – Duas Cobras Cave; 7 – Tunel II Cave; 8 – São Geraldo Cave; 9 – Olho D’água do Cumbra Cave; 10 – Racha Bovina Cave; 11 – Tunel I Cave; 12 – Couve-Flor Cave; 13 – Geraldo Cruz Cave; 14 – Fenda Oblíqua Cave; 15 – Cedro Cave; 16 – Cedrão Cave; 17 – Cedrículo Cave; 18 – Pajeú Cave; 19 – Cristal Cave; 20 – Salobro Cave; 21 – Grota Cave; 22 – Leão Cave; 23 – Cinquentona Cave; 24 – Pedra Escrevidinha Cave.

| **Subclass/Order** | **Family** | **Specie** | **Habitat** | **Quad** | **Tran** | **Add** | **Occurrence** | **Troglomorphic Traits** |
| --- | --- | --- | --- | --- | --- | --- | --- | --- |
| Siluriformes | Heptapteridae | ***Pimelodella* sp1** | Aquatic | 0 | 0 | 1 | 2 | Eyes absence; depigmentation; appendages elongation |
| Acari |  | Acari sp1 | Terrestrial | 0 | 1 | 1 | 5, 23 |  |
| Acari |  | Acari sp2 | Terrestrial | 1 | 0 | 0 | 3 |  |
| Acari |  | Acari sp3 | Terrestrial | 0 | 1 | 1 | 4, 12 |  |
| Acari |  | Acari sp4 | Terrestrial | 0 | 0 | 1 | 6 |  |
| Acari |  | Acari sp5 | Terrestrial | 0 | 0 | 1 | 2 |  |
| Acari |  | Acari sp6 | Terrestrial | 0 | 1 | 0 | 2 |  |
| Amblypygi | Charinidae | *Charinus* sp1 | Terrestrial | 2 | 3 | 3 | 2, 9, 10 |  |
| Amblypygi | Phrynichidae | *Tricodamon* sp1 | Terrestrial | 0 | 0 | 10 | 2, 4, 5, 6, 10, 13, 15, 16, 18, 23 |  |
| Amblypygi | Phrynidae | *Heterophrynus* sp1 | Terrestrial | 0 | 0 | 1 | 20 |  |
| Araneae | Araneidae | Araneidae sp1 | Terrestrial | 0 | 0 | 1 | 20 |  |
| Araneae | Caponidae | **Caponidae sp1** | Terrestrial | 0 | 0 | 1 | 21 | Eyes absence; depigmentation |
| Araneae | Ctenidae | Ctenidae sp1 | Terrestrial | 1 | 2 | 1 | 2, 4 |  |
| Araneae | Ctenidae | Ctenidae sp2 | Terrestrial | 0 | 20 | 1 | 2 |  |
| Araneae | Ctenidae | Ctenidae sp3 | Terrestrial | 3 | 9 | 1 | 2 |  |
| Araneae | Ctenidae | Ctenidae sp4 | Terrestrial | 0 | 2 | 0 | 5, 24 |  |
| Araneae | Ctenidae | Ctenidae sp5 | Terrestrial | 2 | 4 | 1 | 3, 6 |  |
| Araneae | Ctenidae | Ctenidae sp6 | Terrestrial | 1 | 1 | 0 | 4 |  |
| Araneae | Ctenidae | Ctenidae sp7 | Terrestrial | 1 | 0 | 0 | 1 |  |
| Araneae | Ctenidae | Ctenidae sp8 | Terrestrial | 1 | 1 | 0 | 9, 10 |  |
| Araneae | Ctenidae | *Ctenus* sp1 | Terrestrial | 1 | 8 | 5 | 2, 3, 5, 10, 18 |  |
| Araneae | Ctenidae | *Ctenus* sp2 | Terrestrial | 0 | 1 | 0 | 3 |  |
| Araneae | Nesticidae | *Eidmanella pallida* | Terrestrial | 24 | 93 | 3 | 2, 15, 16 |  |
| Araneae | Nesticidae | Nesticidae sp1 | Terrestrial | 2 | 11 | 3 | 2, 4, 16 |  |
| Araneae | Nesticidae | Nesticidae sp2 | Terrestrial | 0 | 0 | 1 | 2 |  |
| Araneae | Nesticidae | Nesticidae sp3 | Terrestrial | 0 | 0 | 1 | 2 |  |
| Araneae | Nesticidae | Nesticidae sp4 | Terrestrial | 0 | 0 | 1 | 2 |  |
| Araneae | Nesticidae | Nesticidae sp5 | Terrestrial | 2 | 0 | 1 | 5, 23 |  |
| Araneae | Nesticidae | Nesticidae sp6 | Terrestrial | 0 | 1 | 0 | 3 |  |
| Araneae | Nesticidae | Nesticidae sp7 | Terrestrial | 10 | 18 | 4 | 1, 4, 7, 9, 10, 11, 12 |  |
| Araneae | Nesticidae | Nesticidae sp8 | Terrestrial | 32 | 72 | 1 | 18, 19 |  |
| Araneae | Nesticidae | Nesticidae sp9 | Terrestrial | 1 | 1 | 3 | 18, 20, 21 |  |
| Araneae | Ochyroceratidae | **Ochyroceratidae sp1** | Terrestrial | 3 | 6 | 1 | 2, 5 |  |
| Araneae | Ochyroceratidae | **Ochyroceratidae sp2** | Terrestrial | 1 | 0 | 1 | 18 | Eyes absence; depigmentation; appendages elongation |
| Araneae | Oecobidae | *Oecobius* sp1 | Terrestrial | 3 | 2 | 0 | 2 | Eyes absence; depigmentation; appendages elongation |
| Araneae | Oonopidae | Oonopidae sp1 | Terrestrial | 0 | 1 | 2 | 3, 16 |  |
| Araneae | Oonopidae | Oonopidae sp2 | Terrestrial | 0 | 2 | 1 | 7, 8 |  |
| Araneae | Oonopidae | Oonopidae sp3 | Terrestrial | 0 | 0 | 1 | 13 |  |
| Araneae | Oonopidae | Oonopidae sp4 | Terrestrial | 0 | 1 | 1 | 20 |  |
| Araneae | Palpimanidae | *Fernandezina* sp1 | Terrestrial | 0 | 1 | 0 | 19 |  |
| Araneae | Palpimanidae | Palpimanidae sp1 | Terrestrial | 0 | 1 | 0 | 3 |  |
| Araneae | Pholcidae | Pholcidae sp1 | Terrestrial | 2 | 13 | 6 | 2, 15, 16, 17, 19, 20, 21 |  |
| Araneae | Pholcidae | Pholcidae sp2 | Terrestrial | 14 | 35 | 15 | 1, 2, 3, 4, 5, 6, 7, 9, 10, 12, 13, 15, 16, 17, 20, 21, 22, 23 |  |
| Araneae | Pholcidae | Pholcidae sp3 | Terrestrial | 15 | 22 | 4 | 3, 6, 8, 9, 10, 11, 12, 22 |  |
| Araneae | Pholcidae | Pholcidae sp4 | Terrestrial | 12 | 10 | 1 | 1, 6, 7, 11, 13, 22 |  |
| Araneae | Pholcidae | Pholcidae sp5 | Terrestrial | 0 | 2 | 0 | 18 |  |
| Araneae | Pholcidae | Pholcidae sp6 | Terrestrial | 0 | 1 | 0 | 18 |  |
| Araneae | Pholcidae | Pholcidae sp7 | Terrestrial | 0 | 3 | 0 | 18 |  |
| Araneae | Pholcidae | *Spermophora* sp1 | Terrestrial | 1 | 0 | 0 | 3 |  |
| Araneae | Prodidomidae | Prodidomidae sp1 | Terrestrial | 0 | 0 | 1 | 6 |  |
| Araneae | Salticidae | Salticidae sp1 | Terrestrial | 0 | 16 | 8 | 1, 2, 3, 4, 5, 6, 7, 8, 9, 11, 18, 24 |  |
| Araneae | Salticidae | Salticidae sp2 | Terrestrial | 0 | 1 | 0 | 10 |  |
| Araneae | Scitodidae | Scitodidae sp1 | Terrestrial | 1 | 0 | 1 | 19, 24 |  |
| Araneae | Sicariidae | *Loxosceles* sp1 | Terrestrial | 44 | 181 | 12 | 1, 2, 3, 4, 5, 7, 8, 10, 11, 12, 16, 17, 19, 23, 24 |  |
| Araneae | Sicariidae | Loxosceles sp2 | Terrestrial | 24 | 92 | 1 | 2, 22 |  |
| Araneae | Sicariidae | Loxosceles sp3 | Terrestrial | 1 | 2 | 2 | 2, 3 |  |
| Araneae | Sicariidae | Loxosceles sp4 | Terrestrial | 2 | 11 | 2 | 18, 19 |  |
| Araneae | Sicariidae | Sicariidae sp1 | Terrestrial | 1 | 5 | 1 | 2, 4, 6, 10 |  |
| Araneae | Sicariidae | Sicariidae sp2 | Terrestrial | 1 | 0 | 0 | 3 |  |
| Araneae | Sicariidae | Sicariidae sp3 | Terrestrial | 1 | 2 | 1 | 8 |  |
| Araneae | Telemidae | **Telemidae sp1** | Terrestrial | 0 | 0 | 1 | 20 | Eyes absence; depigmentation |
| Araneae | Tetragnathidae | *Leucauge* sp1 | Terrestrial | 1 | 22 | 14 | 1, 2, 3, 4, 5, 7, 8, 10, 11, 12, 13, 15, 19, 22, 24 |  |
| Araneae | Theraphosidae | *Lasiodora* sp1 | Terrestrial | 0 | 0 | 1 | 20 |  |
| Araneae | Theraphosidae | Theraphosidae sp1 | Terrestrial | 0 | 0 | 1 | 2 |  |
| Araneae | Theridiidae | *Criptochaea* sp1 | Terrestrial | 2 | 4 | 1 | 2, 22 |  |
| Araneae | Theridiidae | Theridiidae sp1 | Terrestrial | 0 | 0 | 1 | 3 |  |
| Araneae | Theridiidae | Theridiidae sp2 | Terrestrial | 0 | 1 | 1 | 3, 6 |  |
| Araneae | Trechaleidae | Trechaleidae sp1 | Terrestrial | 0 | 4 | 4 | 2, 15, 16, 19, 20 |  |
| Araneae | Trechaleidae | Trechaleidae sp2 | Terrestrial | 0 | 1 | 1 | 3, 22 |  |
| Araneae | Trechaleidae | Trechaleidae sp3 | Terrestrial | 0 | 8 | 3 | 4, 6, 13, 23 |  |
| Araneae | Trechaleidae | Trechaleidae sp4 | Terrestrial | 3 | 11 | 6 | 1, 7, 8, 10, 11, 12 |  |
| Araneae | Ulboridae | Uloboridae sp1 | Terrestrial | 2 | 4 | 2 | 3, 6, 11 |  |
| Araneae | Ulboridae | Uloboridae sp2 | Terrestrial | 1 | 0 | 3 | 1, 7, 12 |  |
| Araneae | Ulboridae | Uloboridae sp3 | Terrestrial | 0 | 0 | 2 | 9, 11 |  |
| Araneae | Ulboridae | Uloboridae sp4 | Terrestrial | 1 | 2 | 1 | 9, 10, 21 |  |
| Araneae |  | Araneae sp3 | Terrestrial | 0 | 0 | 1 | 3 |  |
| Araneae |  | Araneae sp4 | Terrestrial | 0 | 1 | 0 | 3 |  |
| Ixodida | Argasidae | *Ornithodoros* sp1 | Terrestrial | 0 | 1 | 3 | 2, 14, 21, 22 |  |
| Ixodida |  | Ixodida sp1 | Terrestrial | 0 | 0 | 1 | 18 |  |
| Mesostigmata | Eviphididae | Eviphididae sp1 | Terrestrial | 1 | 0 | 1 | 20, 22 |  |
| Mesostigmata | Laelapidae | *Gaeolaelaps* sp1 | Terrestrial | 0 | 0 | 1 | 2 |  |
| Mesostigmata | Reginacharlottiidae | *Reginacharlottia* sp1 | Terrestrial | 0 | 0 | 1 | 2 |  |
| Mesostigmata |  | Mesostigmata sp1 | Terrestrial | 1 | 0 | 0 | 18 |  |
| Mesostigmata |  | Mesostigmata sp2 | Terrestrial | 13 | 35 | 1 | 20, 21, 22 |  |
| Opiliones | Escadabiidae | **Escadabiidae sp1** | Terrestrial | 0 | 5 | 2 | 2, 16, 22 | Eyes reduction; depigmentation; appendages elongation |
| Opiliones | Escadabiidae | **Escadabiidae sp2** | Terrestrial | 0 | 0 | 2 | 20, 21 | Eyes reduction; depigmentation; appendages elongation |
| Opiliones | Gonyleptidae | *Eusarcus cf. cavernicola* | Terrestrial | 4 | 20 | 3 | 2, 4, 5 |  |
| Opiliones | Gonyleptidae | *Eusarcus* sp1 | Terrestrial | 3 | 12 | 4 | 2, 3, 10, 19 |  |
| Opiliones | Gonyleptidae | ***Eusarcus* sp2** | Terrestrial | 0 | 4 | 0 | 2 | Eyes reduction; depigmentation; appendages elongation |
| Opiliones | Sclerosomatidae | Sclerosomatidae sp1 | Terrestrial | 0 | 2 | 2 | 1, 4, 22 |  |
| Opiliones |  | Opiliones sp1 | Terrestrial | 1 | 0 | 0 | 2 |  |
| Palpigradi | Eukoeneniidae | **Eukoenenia sp1** | Terrestrial | 2 | 2 | 2 | 2, 22 | Appendages elongation; developed sensory organs |
| Palpigradi | Eukoeneniidae | **Eukoenenia sp2** | Terrestrial | 0 | 0 | 1 | 2 | Appendages elongation; developed sensory organs |
| Pseudoscorpiones | Chernetiidae | Chernetiidae sp1 | Terrestrial | 2 | 1 | 0 | 2 |  |
| Pseudoscorpiones | Chthoniidae | *Pseudochthonius aware* | Terrestrial | 11 | 29 | 2 | 2, 15, 22 |  |
| Pseudoscorpiones | Chthoniidae | ***Pseudochthonius* sp2** | Terrestrial | 0 | 0 | 1 | 5 | Eyes absence; depigmentation; appendages elongation |
| Pseudoscorpiones | Garypidae | Garypidae sp1 | Terrestrial | 22 | 52 | 4 | 6, 9, 10, 13, 18, 19, 23, 24 |  |
| Pseudoscorpiones | Ideoroncidae | **Ideoroncidae sp1** | Terrestrial | 0 | 1 | 1 | 6, 22 | Eyes absence; depigmentation; appendages elongation |
| Pseudoscorpiones |  | Garypoidea sp1 | Terrestrial | 3 | 6 | 2 | 20, 21 |  |
| Pseudoscorpiones |  | Pseudoscorpiones sp1 | Terrestrial | 1 | 4 | 2 | 7, 8 |  |
| Sarcoptiformes | Euphthiracaridae | Euphthiracaridae sp1 | Terrestrial | 0 | 0 | 1 | 2 |  |
| Scorpiones | Buthidae | *Tityus serrulatus* | Terrestrial | 0 | 0 | 1 | 7 |  |
| Trombidiformes | Erythraeidae | *Callidosoma* sp1 | Terrestrial | 2 | 0 | 1 | 2, 15 |  |
| Trombidiformes | Macronyssidae | Macronyssidae sp1 | Terrestrial | 5 | 6 | 0 | 2, 17, 22, 24 |  |
| Trombidiformes | Rhagidiidae | Rhagidiidae sp1 | Terrestrial | 2 | 1 | 1 | 2, 15 |  |
| Sphaeriida | Sphaeriidae | ***Eupera* sp1** | Aquatic | 0 | 0 | 1 | 2 | Depigmentation |
| Scolopendromorpha | Scolopendride | Sterropristinae sp1 | Terrestrial | 0 | 0 | 2 | 16, 23 |  |
| Scutigeromorpha | Pselliodidae | Pselliodidae sp1 | Terrestrial | 0 | 1 | 0 | 3 |  |
| Haplotaxida |  | **Lumbricina sp1** | Terrestrial | 8 | 18 | 2 | 2, 4, 20, 21, 22 | Depigmentation; body elongarion |
| Haplotaxida |  | Lumbricina sp2 | Terrestrial | 4 | 36 | 1 | 2 |  |
| Haplotaxida |  | Lumbricina sp3 | Terrestrial | 0 | 0 | 1 | 2 |  |
| Haplotaxida |  | Lumbricina sp4 | Terrestrial | 0 | 2 | 0 | 2 |  |
| Entomobryomorpha | Entomobryidae | *Lepidocyrtinus* sp1 | Terrestrial | 1 | 1 | 0 | 2, 16 |  |
| Entomobryomorpha | Entomobryidae | *Lepidocyrtinus* sp2 | Terrestrial | 4 | 3 | 2 | 3, 6, 10, 13 |  |
| Entomobryomorpha | Paronellidae | **Paronellidae sp1** | Terrestrial | 112 | 576 | 4 | 2, 5, 15, 16, 20, 21, 22, 24 | Eyes absence; depigmentation; appendages elongation |
| Entomobryomorpha | Paronellidae | Paronellidae sp2 | Terrestrial | 8 | 11 | 1 | 2, 21 |  |
| Entomobryomorpha | Paronellidae | Paronellidae sp3 | Terrestrial | 0 | 0 | 1 | 3 |  |
| Entomobryomorpha | Paronellidae | Paronellidae sp4 | Terrestrial | 15 | 22 | 3 | 1, 3, 4, 6, 9 |  |
| Entomobryomorpha | Paronellidae | Paronellidae sp5 | Terrestrial | 0 | 1 | 0 | 18 |  |
| Poduromorpha |  | **Poduromorpha sp1** | Terrestrial | 0 | 0 | 1 | 2 | Eyes absence; depigmentation; appendages elongation |
| Symphypleona | Arrhopalitidae | **Arrhopalitidae sp1** | Terrestrial | 2 | 0 | 1 | 2 | Eyes absence; depigmentation; appendages elongation |
| Symphypleona | Arrhopalitidae | Arrhopalitidae sp2 | Terrestrial | 0 | 0 | 1 | 22 |  |
| Spirostreptida | Pseudonannolenidae | Pseudonannolene sp1 | Terrestrial | 17 | 85 | 2 | 2, 4 |  |
| Spirostreptida | Pseudonannolenidae | Pseudonannolene sp2 | Terrestrial | 7 | 16 | 1 | 2, 3 |  |
| Spirostreptida | Pseudonannolenidae | Pseudonannolene sp3 | Terrestrial | 7 | 11 | 2 | 2, 4 |  |
| Spirostreptida | Pseudonannolenidae | Pseudonannolene sp4 | Terrestrial | 0 | 2 | 0 | 2 |  |
| Spirostreptida | Pseudonannolenidae | Pseudonannolene sp5 | Terrestrial | 2 | 0 | 1 | 20, 21 |  |
| Spirostreptida |  | ***Phaneromerium cavernicolum*** | Terrestrial | 12 | 29 | 4 | 2, 4, 9, 14, 18 | Eyes absence; depigmentation; appendages elongation |
| Spirostreptida |  | Spirostreptida sp1 | Terrestrial | 1 | 0 | 0 | 2 |  |
| Littorinimorpha | Tateidae | ***Potamolithus* sp1** | Terrestrial | 0 | 0 | 1 | 2 | Depigmentation |
|  |  | Gastropoda sp1 | Terrestrial | 0 | 1 | 1 | 2, 9 |  |
|  |  | Gastropoda sp2 | Terrestrial | 0 | 1 | 0 | 10 |  |
| Blattodea | Blattidae | Blattellidae sp1 | Terrestrial | 0 | 0 | 1 | 2 |  |
| Blattodea | Blattidae | Blattellidae sp2 | Terrestrial | 6 | 0 | 1 | 2, 17 |  |
| Blattodea | Blattidae | **Blattellidae sp3** | Terrestrial | 1 | 0 | 3 | 18, 20, 21 | Eyes absence; depigmentation |
| Blattodea | Polyphagidae | Polyphagidae sp1 | Terrestrial | 0 | 1 | 0 | 2 |  |
| Blattodea |  | Blattodea sp1 | Terrestrial | 0 | 1 | 0 | 3 |  |
| Blattodea |  | Blattodea sp2 | Terrestrial | 0 | 0 | 1 | 4 |  |
| Blattodea |  | Blattodea sp3 | Terrestrial | 1 | 0 | 1 | 8 |  |
| Blattodea |  | Blattodea sp4 | Terrestrial | 0 | 2 | 2 | 9, 13, 21 |  |
| Blattodea |  | Blattodea sp5 | Terrestrial | 0 | 1 | 0 | 18 |  |
| Blattodea |  | Isoptera sp1 | Terrestrial | 8 | 109 | 4 | 7, 9, 10, 13, 17, 20, 21, 22 |  |
| Coleoptera | Carabidae | Carabidae sp1 | Terrestrial | 5 | 86 | 1 | 2 |  |
| Coleoptera | Carabidae | Carabidae sp10 | Terrestrial | 1 | 0 | 0 | 9 |  |
| Coleoptera | Carabidae | Carabidae sp11 | Terrestrial | 2 | 8 | 1 | 18, 19 |  |
| Coleoptera | Carabidae | Carabidae sp2 | Terrestrial | 3 | 8 | 3 | 2, 16, 17 |  |
| Coleoptera | Carabidae | Carabidae sp3 | Terrestrial | 3 | 4 | 2 | 2, 20 |  |
| Coleoptera | Carabidae | Carabidae sp4 | Terrestrial | 0 | 0 | 1 | 2 |  |
| Coleoptera | Carabidae | Carabidae sp5 | Terrestrial | 0 | 5 | 1 | 2 |  |
| Coleoptera | Carabidae | Carabidae sp7 | Terrestrial | 0 | 2 | 1 | 5, 24 |  |
| Coleoptera | Carabidae | Carabidae sp8 | Terrestrial | 0 | 0 | 1 | 3 |  |
| Coleoptera | Carabidae | Carabidae sp9 | Terrestrial | 2 | 9 | 1 | 1, 4 |  |
| Coleoptera | Carabidae | ***Clivina* sp1** | Terrestrial | 4 | 8 | 1 | 2, 4 | Eyes absence; depigmentation; appendages elongation |
| Coleoptera | Carabidae | ***Coarazuphium tessai*** | Terrestrial | 0 | 0 | 1 | 2 | Eyes absence; depigmentation; appendages elongation |
| Coleoptera | Carabidae | *Colliuris* sp1 | Terrestrial | 0 | 0 | 1 | 21 |  |
| Coleoptera | Chrysomelidae | Chrysomelidae sp1 | Terrestrial | 0 | 3 | 3 | 2, 8, 17, 21 |  |
| Coleoptera | Curculionidae | Curculionidae sp1 | Terrestrial | 0 | 1 | 2 | 3, 10, 13 |  |
| Coleoptera | Dermestidae | Dermestidae larva sp1 | Terrestrial | 93 | 41 | 0 | 7, 8, 16, 17, 18, 19 |  |
| Coleoptera | Dermestidae | Dermestidae larva sp2 | Terrestrial | 0 | 1 | 1 | 23 |  |
| Coleoptera | Erotylidae | *Ischyrus* sp1 | Terrestrial | 0 | 0 | 1 | 21 |  |
| Coleoptera | Helodidae | Helodidae sp1 | Terrestrial | 0 | 1 | 0 | 9 |  |
| Coleoptera | Hydroscaphidae | Hydroscaphidae sp1 | Terrestrial | 0 | 1 | 0 | 21 |  |
| Coleoptera | Leiodidae | Leiodidae sp1 | Terrestrial | 1 | 6 | 4 | 2, 4, 5, 15, 16, 17, 22 |  |
| Coleoptera | Scarabaeidae | Scarabaeidae sp1 | Terrestrial | 0 | 0 | 1 | 21 |  |
| Coleoptera | Scarabaeidae | *Uroxys* sp1 | Terrestrial | 0 | 0 | 2 | 3, 14 |  |
| Coleoptera | Staphylinidae | Staphylinidae sp1 | Terrestrial | 59 | 79 | 3 | 2, 5, 15, 22, 23, 24 |  |
| Coleoptera | Staphylinidae | Staphylinidae sp2 | Terrestrial | 0 | 1 | 0 | 2 |  |
| Coleoptera | Staphylinidae | Staphylinidae sp3 | Terrestrial | 0 | 0 | 1 | 2 |  |
| Coleoptera | Staphylinidae | Staphylinidae sp4 | Terrestrial | 3 | 14 | 0 | 1, 3, 4, 9 |  |
| Coleoptera | Staphylinidae | Staphylinidae sp5 | Terrestrial | 3 | 0 | 0 | 9, 10 |  |
| Coleoptera | Staphylinidae | Staphylinidae sp6 | Terrestrial | 2 | 2 | 1 | 20, 21 |  |
| Coleoptera | Tenebrionidae | Tenebrionidae sp1 | Terrestrial | 0 | 1 | 1 | 2, 8 |  |
| Coleoptera | Tenebrionidae | Tenebrionidae sp2 | Terrestrial | 0 | 0 | 1 | 20 |  |
| Coleoptera | Tenebrionidae | *Zophobas* sp1 | Terrestrial | 1 | 8 | 1 | 18 |  |
| Coleoptera |  | Coleoptera larva sp1 | Terrestrial | 0 | 1 | 0 | 4 |  |
| Coleoptera |  | Coleoptera larva sp2 | Terrestrial | 0 | 1 | 0 | 8 |  |
| Coleoptera |  | Coleoptera larva sp3 | Terrestrial | 0 | 1 | 0 | 9 |  |
| Coleoptera |  | Coleoptera larva sp4 | Terrestrial | 2 | 0 | 0 | 9 |  |
| Coleoptera |  | Coleoptera sp5 | Terrestrial | 1 | 0 | 0 | 10 |  |
| Dermaptera | Anisolabididae | Anisolabididae sp1 | Terrestrial | 0 | 0 | 1 | 21 |  |
| Dermaptera | Anisolabididae | Anisolabididae sp2 | Terrestrial | 1 | 0 | 1 | 15, 17 |  |
| Diptera | Anthomyiidae | Anthomyiidae sp1 | Terrestrial | 1 | 1 | 0 | 9 |  |
| Diptera | Cecidomyiidae | Cecidomyiidae sp1 | Terrestrial | 1 | 0 | 1 | 1, 23 |  |
| Diptera | Cecidomyiidae | Cecidomyiidae sp2 | Terrestrial | 2 | 3 | 0 | 21 |  |
| Diptera | Cecidomyiidae | Cecidomyiidae sp3 | Terrestrial | 0 | 0 | 2 | 1, 23 |  |
| Diptera | Ceratopogonidae | Ceratopogonidae sp1 | Terrestrial | 0 | 0 | 1 | 4 |  |
| Diptera | Chironomidae | Chironomidae sp1 | Terrestrial | 0 | 0 | 1 | 1 |  |
| Diptera | Dixidae | Dixidae sp1 | Terrestrial | 1 | 0 | 0 | 5 |  |
| Diptera | Dolichopodidae | Dolichopodidae sp1 | Terrestrial | 0 | 0 | 1 | 1 |  |
| Diptera | Dolichopodidae | Dolichopodidae sp2 | Terrestrial | 0 | 27 | 2 | 7, 9, 24 |  |
| Diptera | Dolichopodidae | Dolichopodidae sp3 | Terrestrial | 0 | 0 | 1 | 18 |  |
| Diptera | Dolichopodidae | Dolichopodidae sp4 | Terrestrial | 1 | 2 | 0 | 18 |  |
| Diptera | Dolichopodidae | Dolichopodidae sp5 | Terrestrial | 1 | 0 | 0 | 22 |  |
| Diptera | Dolichopodidae | Dolichopodidae sp6 | Terrestrial | 0 | 7 | 0 | 24 |  |
| Diptera | Drosophilidae | Drosophilidae sp1 | Terrestrial | 0 | 1 | 2 | 9, 17 |  |
| Diptera | Drosophilidae | Drosophilidae sp2 | Terrestrial | 6 | 35 | 2 | 20, 21 |  |
| Diptera | Milichiidae | Milichiidae sp1 | Terrestrial | 0 | 1 | 0 | 22 |  |
| Diptera | Muscidae | Muscidae sp1 | Terrestrial | 0 | 1 | 0 | 2 |  |
| Diptera | Muscidae | Muscidae sp2 | Terrestrial | 0 | 0 | 1 | 2 |  |
| Diptera | Muscidae | Muscidae sp3 | Terrestrial | 0 | 0 | 1 | 9 |  |
| Diptera | Mycetophilidae | Mycetophilidae sp1 | Terrestrial | 0 | 0 | 1 | 3 |  |
| Diptera | Phoridae | *Conicera* sp1 | Terrestrial | 0 | 0 | 1 | 2 |  |
| Diptera | Phoridae | *Conicera* sp2 | Terrestrial | 0 | 1 | 0 | 2 |  |
| Diptera | Phoridae | *Conicera* sp3 | Terrestrial | 1 | 0 | 1 | 4, 5 |  |
| Diptera | Phoridae | *Conicera* sp4 | Terrestrial | 1 | 0 | 0 | 7 |  |
| Diptera | Phoridae | Phoridae sp1 | Terrestrial | 0 | 0 | 1 | 2 |  |
| Diptera | Phoridae | Phoridae sp2 | Terrestrial | 1 | 0 | 0 | 1 |  |
| Diptera | Phoridae | Phoridae sp3 | Terrestrial | 2 | 1 | 0 | 18 |  |
| Diptera | Phoridae | Phoridae sp4 | Terrestrial | 0 | 2 | 2 | 20, 21 |  |
| Diptera | Phoridae | Phoridae sp5 | Terrestrial | 0 | 5 | 1 | 15, 16 |  |
| Diptera | Psychodidae | *Lutzomya* sp1 | Terrestrial | 2 | 2 | 0 | 2, 4, 17 |  |
| Diptera | Psychodidae | *Lutzomya* sp2 | Terrestrial | 24 | 19 | 11 | 1, 2, 4, 5, 7, 8, 9, 10, 11, 12, 15, 16, 17, 18, 19, 20, 21, 22 |  |
| Diptera | Psychodidae | *Lutzomya* sp3 | Terrestrial | 0 | 2 | 0 | 2 |  |
| Diptera | Psychodidae | *Lutzomya* sp4 | Terrestrial | 0 | 2 | 0 | 2 |  |
| Diptera | Psychodidae | *Lutzomya* sp5 | Terrestrial | 46 | 34 | 0 | 2 |  |
| Diptera | Psychodidae | Psychodidae sp1 | Terrestrial | 0 | 0 | 2 | 2, 11 |  |
| Diptera | Psychodidae | Psychodidae sp2 | Terrestrial | 0 | 1 | 1 | 20, 22 |  |
| Diptera | Sciaridae | Sciaridae sp1 | Terrestrial | 5 | 5 | 2 | 1, 2, 4, 14, 16 |  |
| Diptera | Sciaridae | Sciaridae sp2 | Terrestrial | 2 | 2 | 1 | 5 |  |
| Diptera | Sciaridae | Sciaridae sp3 | Terrestrial | 0 | 0 | 1 | 5 |  |
| Diptera | Sciaridae | Sciaridae sp4 | Terrestrial | 0 | 0 | 1 | 4 |  |
| Diptera | Stratiomyidae | Stratiomyidae sp1 | Terrestrial | 0 | 0 | 1 | 20 |  |
| Diptera | Tipulidae | Tipulidae sp1 | Terrestrial | 0 | 0 | 1 | 20 |  |
| Diptera |  | Diptera larva sp1 | Terrestrial | 0 | 20 | 1 | 2 |  |
| Diptera |  | Diptera larva sp2 | Terrestrial | 0 | 1 | 1 | 2 |  |
| Diptera |  | Diptera larva sp3 | Terrestrial | 1 | 0 | 0 | 4 |  |
| Diptera |  | Diptera larva sp4 | Terrestrial | 0 | 1 | 0 | 1 |  |
| Diptera |  | Diptera larva sp5 | Terrestrial | 0 | 1 | 0 | 20 |  |
| Diptera |  | Diptera pupa sp1 | Terrestrial | 0 | 0 | 1 | 5 |  |
| Diptera |  | Diptera sp1 | Terrestrial | 0 | 1 | 0 | 1 |  |
| Diptera |  | Diptera larva sp6 | Terrestrial | 0 | 0 | 1 | 4 |  |
| Ephemeroptera |  | Ephemeroptera sp1 | Aquatic | 0 | 0 | 1 | 2 |  |
| Hemiptera | Cixiidae | Cixiidae sp1 | Terrestrial | 2 | 5 | 2 | 3, 10, 24 |  |
| Hemiptera | Cixiidae | Cixiidae sp2 | Terrestrial | 0 | 6 | 1 | 6, 9 |  |
| Hemiptera | Cixiidae | **Cixiidae sp3** | Terrestrial | 4 | 6 | 2 | 3, 20 | Eyes absence; depigmentation |
| Hemiptera | Kinnaridae | Kinnaridae sp2 | Terrestrial | 0 | 0 | 1 | 21 |  |
| Hemiptera | Lygaeidae | Lygaeidae sp1 | Terrestrial | 1 | 5 | 0 | 3, 6, 10 |  |
| Hemiptera | Lygaeidae | Lygaeidae sp2 | Terrestrial | 0 | 0 | 1 | 2 |  |
| Hemiptera | Reduviidae | Emesinae sp1 | Terrestrial | 2 | 0 | 2 | 2, 16, 17 |  |
| Hemiptera | Reduviidae | Emesinae sp2 | Terrestrial | 1 | 2 | 2 | 2, 3, 6, 20 |  |
| Hemiptera | Reduviidae | Emesinae sp3 | Terrestrial | 0 | 1 | 0 | 10 |  |
| Hemiptera | Reduviidae | *Zelurus* sp1 | Terrestrial | 18 | 76 | 10 | 2, 3, 5, 6, 7, 8, 9, 10, 11, 12, 14, 15, 16, 17, 19, 22, 23, 24 |  |
| Hemiptera | Veliidae | Veliidae sp1 | Terrestrial | 4 | 15 | 5 | 2, 4, 9, 10, 14, 20, 22 |  |
| Hemiptera |  | Coccoidea sp1 | Terrestrial | 1 | 0 | 0 | 9 |  |
| Hemiptera |  | Coccoidea sp2 | Terrestrial | 1 | 1 | 1 | 20, 22 |  |
| Hemiptera |  | Gymnocerata sp1 | Terrestrial | 0 | 0 | 1 | 10 |  |
| Hemiptera |  | Hemiptera sp1 | Terrestrial | 1 | 0 | 1 | 10, 11 |  |
| Hemiptera |  | Homoptera sp1 | Terrestrial | 0 | 1 | 0 | 10 |  |
| Hymenoptera | Apidae | *Trigona* sp1 | Terrestrial | 1 | 0 | 0 | 18 |  |
| Hymenoptera | Braconidae | Braconidae sp1 | Terrestrial | 0 | 0 | 1 | 7 |  |
| Hymenoptera | Braconidae | Braconidae sp2 | Terrestrial | 1 | 0 | 0 | 13 |  |
| Hymenoptera | Eulophidae | Eulophidae sp1 | Terrestrial | 1 | 0 | 0 | 5 |  |
| Hymenoptera | Formicidae | Formicidae sp1 | Terrestrial | 0 | 0 | 1 | 3 |  |
| Hymenoptera | Formicidae | Formicidae sp2 | Terrestrial | 0 | 1 | 1 | 3 |  |
| Hymenoptera | Formicidae | Formicidae sp3 | Terrestrial | 55 | 111 | 6 | 1, 3, 5, 6, 8, 10, 11, 12, 22, 23, 24 |  |
| Hymenoptera | Formicidae | Formicidae sp4 | Terrestrial | 1 | 1 | 4 | 6, 7, 9, 11 |  |
| Hymenoptera | Formicidae | Formicidae sp5 | Terrestrial | 0 | 0 | 1 | 9 |  |
| Hymenoptera | Formicidae | Formicidae sp6 | Terrestrial | 0 | 4 | 0 | 9 |  |
| Hymenoptera | Formicidae | Formicidae sp7 | Terrestrial | 0 | 2 | 0 | 10 |  |
| Hymenoptera | Formicidae | Formicidae sp8 | Terrestrial | 0 | 1 | 0 | 14 |  |
| Hymenoptera | Formicidae | Formicidae sp9 | Terrestrial | 0 | 1 | 1 | 18, 19 |  |
| Hymenoptera | Formicidae | *Gnamptogenys* sp1 | Terrestrial | 0 | 0 | 1 | 2 |  |
| Hymenoptera | Formicidae | *Hypoponera* sp1 | Terrestrial | 4 | 2 | 3 | 2, 11, 17 |  |
| Hymenoptera | Formicidae | *Hypoponera* sp2 | Terrestrial | 0 | 0 | 1 | 3 |  |
| Hymenoptera | Formicidae | *Labidus* sp1 | Terrestrial | 0 | 4 | 2 | 20, 21, 22 |  |
| Hymenoptera | Formicidae | *Odontomachus* sp1 | Terrestrial | 2 | 7 | 1 | 2 |  |
| Hymenoptera | Formicidae | *Pheidole* sp1 | Terrestrial | 0 | 0 | 3 | 3, 5, 16 |  |
| Hymenoptera | Formicidae | Dolichoderinae sp1 | Terrestrial | 3 | 7 | 2 | 15, 17, 22 |  |
| Hymenoptera | Ichneumonidae | Ichneumonidae sp1 | Terrestrial | 1 | 1 | 1 | 9, 22 |  |
| Hymenoptera | Trichogrammatidae | Trichogrammatidae sp1 | Terrestrial | 0 | 1 | 0 | 21 |  |
| Hymenoptera |  | Chalcidoidea sp1 | Terrestrial | 1 | 0 | 1 | 6 |  |
| Hymenoptera |  | Chalcidoidea sp2 | Terrestrial | 1 | 0 | 0 | 9 |  |
| Hymenoptera |  | Hymenoptera sp1 | Terrestrial | 12 | 4 | 0 | 4 |  |
| Hymenoptera |  | Hymenoptera sp2 | Terrestrial | 1 | 1 | 0 | 8 |  |
| Lepidoptera | Geometridae | Geometridae sp1 | Terrestrial | 0 | 0 | 1 | 20 |  |
| Lepidoptera | Noctuidae | Noctuidae sp1 | Terrestrial | 2 | 1 | 6 | 4, 15, 16, 18, 20, 22, 23 |  |
| Lepidoptera | Pyralidae | Pyralidae sp1 | Terrestrial | 1 | 7 | 8 | 1, 4, 8, 12, 13, 18, 20, 23, 24 |  |
| Lepidoptera | Tineidae | Tineidae sp1 | Terrestrial | 2 | 32 | 5 | 1, 2, 3, 4, 7, 8, 11, 15, 16, 22 |  |
| Lepidoptera | Tineidae | Tineidae sp2 | Terrestrial | 0 | 19 | 1 | 2 |  |
| Lepidoptera | Tineidae | Tineidae sp3 | Terrestrial | 0 | 3 | 0 | 2 |  |
| Lepidoptera | Tineidae | Tineidae sp4 | Terrestrial | 0 | 4 | 4 | 6, 9, 10, 13 |  |
| Lepidoptera |  | Lepidoptera larva sp1 | Terrestrial | 0 | 1 | 0 | 4 |  |
| Lepidoptera |  | Lepidoptera larva sp2 | Terrestrial | 0 | 1 | 0 | 1 |  |
| Lepidoptera |  | Lepidoptera sp3 | Terrestrial | 0 | 0 | 1 | 20 |  |
| Neuroptera | Mantispidae | Mantispidae sp1 | Terrestrial | 0 | 0 | 1 | 9 |  |
| Neuroptera | Myrmeleontidae | Myrmeleontidae sp1 | Terrestrial | 3 | 14 | 1 | 8, 11, 22, 24 |  |
| Odonata | Gomphidae | Gomphidae sp1 | Terrestrial | 0 | 0 | 1 | 2 |  |
| Odonata |  | Odonota larva sp1 | Terrestrial | 0 | 0 | 1 | 1 |  |
| Orthoptera | Gryllidae | Gryllinae sp1 | Terrestrial | 0 | 0 | 1 | 20 |  |
| Orthoptera | Mogoplistidae | Mogoplistidae sp1 | Terrestrial | 0 | 1 | 1 | 14, 17 |  |
| Orthoptera | Mogoplistidae | Mogoplistidae sp2 | Terrestrial | 3 | 9 | 1 | 18, 20, 22 |  |
| Orthoptera | Phalangopsidae | *Eidmanacris* sp1 | Terrestrial | 4 | 9 | 6 | 1, 3, 4, 7, 9, 10 |  |
| Orthoptera | Phalangopsidae | *Eidmanacris* sp2 | Terrestrial | 0 | 2 | 0 | 22 |  |
| Orthoptera | Phalangopsidae | *Eidmanacris* sp3 | Terrestrial | 3 | 8 | 1 | 20, 21 |  |
| Orthoptera | Phalangopsidae | ***Endecous* sp1** | Terrestrial | 9 | 62 | 12 | 1, 2, 3, 4, 5, 6, 7, 8, 9, 10, 12, 23, 24 | Eyes reduced; depigmentation; appendages elongation |
| Orthoptera |  | Acridomorpha sp1 | Terrestrial | 0 | 0 | 1 | 16 |  |
| Orthoptera |  | Ensifera sp1 | Terrestrial | 1 | 0 | 0 | 13 |  |
| Orthoptera |  | Ensifera sp2 | Terrestrial | 1 | 0 | 0 | 9 |  |
| Orthoptera |  | Ensifera sp3 | Terrestrial | 1 | 2 | 0 | 21 |  |
| Psocoptera | Liposcelididae | Liposcelididae sp1 | Terrestrial | 1 | 0 | 0 | 18 |  |
| Psocoptera | Psyllipsocidae | Psyllipsocidae sp1 | Terrestrial | 3 | 9 | 2 | 2, 15, 16, 17, 22 |  |
| Psocoptera | Psyllipsocidae | Psyllipsocidae sp2 | Terrestrial | 12 | 4 | 0 | 2 |  |
| Psocoptera | Psyllipsocidae | Psyllipsocidae sp3 | Terrestrial | 1 | 0 | 0 | 3 |  |
| Psocoptera | Psyllipsocidae | Psyllipsocidae sp4 | Terrestrial | 1 | 0 | 0 | 3 |  |
| Psocoptera | Psyllipsocidae | Psyllipsocidae sp5 | Terrestrial | 41 | 30 | 5 | 1, 4, 6, 7, 8, 14, 23, 24 |  |
| Psocoptera | Psyllipsocidae | Psyllipsocidae sp6 | Terrestrial | 3 | 12 | 0 | 6, 10 |  |
| Psocoptera | Psyllipsocidae | Psyllipsocidae sp7 | Terrestrial | 11 | 13 | 2 | 18, 19, 21 |  |
| Psocoptera |  | *Neotrogla* sp1 | Terrestrial | 4 | 7 | 2 | 2, 16, 22, 23 |  |
| Psocoptera |  | *Neotrogla* sp2 | Terrestrial | 3 | 11 | 2 | 1, 7, 8, 18 |  |
| Psocoptera |  | *Neotrogla* sp3 | Terrestrial | 0 | 1 | 0 | 18 |  |
| Amphipoda | Artesiidae | ***Spelaeogammarus santanensis*** | Aquatic | 0 | 0 | 1 | 2 | Eyes absence; depigmentation; appendages elongation |
| Isopoda | Armadillidae | Armadillidae sp1 | Terrestrial | 0 | 0 | 1 | 8 |  |
| Isopoda | Armadillidae | *Ctenorillo* sp1 | Terrestrial | 0 | 1 | 0 | 10 |  |
| Isopoda | Armadillidae | *Venezillo* sp1 | Terrestrial | 0 | 0 | 1 | 8 |  |
| Isopoda | Dubioniscidae | Dubioniscidae sp1 | Terrestrial | 0 | 0 | 1 | 10 |  |
| Isopoda | Dubioniscidae | *Novamundoniscus* sp1 | Terrestrial | 2 | 4 | 0 | 16, 17 |  |
| Isopoda | Philosciidae | *Atlantoscia* sp1 | Terrestrial | 0 | 1 | 0 | 17 |  |
| Isopoda | Philosciidae | *Atlantoscia* sp2 | Terrestrial | 0 | 0 | 1 | 21 |  |
| Isopoda | Philosciidae | **Philosciidae sp1** | Terrestrial | 0 | 0 | 1 | 18 | Eyes absence; depigmentation; appendages elongation |
| Isopoda | Plathyarthridae | **Plathyarthridae sp1** | Terrestrial | 2 | 11 | 0 | 2 | Eyes absence; depigmentation; appendages elongation |
| Isopoda | Plathyarthridae | ***Trichorhina* sp1** | Terrestrial | 3 | 3 | 2 | 5, 15, 16, 20 | Eyes absence; depigmentation; appendages elongation |
| Isopoda | Plathyarthridae | *Trichorhina* sp2 | Terrestrial | 0 | 1 | 0 | 20 |  |
| Isopoda | Styloniscidae | ***Chaimowiczia tatus*** | Terrestrial | 0 | 0 | 1 | 2 | Eyes absence; depigmentation; appendages elongation |
| Isopoda | Styloniscidae | ***Pectenoniscus santanensis*** | Terrestrial | 67 | 141 | 1 | 2, 15, 16, 22 | Eyes absence; depigmentation; appendages elongation |
| Isopoda | Styloniscidae | *Pectenoniscus* sp2 | Terrestrial | 3 | 0 | 1 | 5, 10 |  |
| Isopoda | Styloniscidae | ***Pectenoniscus* sp3** | Terrestrial | 0 | 1 | 0 | 22 | Eyes absence; depigmentation; appendages elongation |
| Isopoda | Styloniscidae | ***Xangoniscus paiabare*** | Terrestrial | 1 | 61 | 5 | 2, 5, 14, 22 | Eyes absence; depigmentation; appendages elongation |
| Isopoda | Styloniscidae | **Styloniscidae sp1** | Terrestrial | 3 | 8 | 1 | 2 | Eyes absence; depigmentation; appendages elongation |
| Isopoda | Styloniscidae | **Styloniscidae sp2** | Terrestrial | 2 | 21 | 1 | 5, 11 | Eyes absence; depigmentation; appendages elongation |
| Isopoda |  | **Calabozoidea sp1** | Terrestrial | 1 | 0 | 0 | 20 | Eyes absence; depigmentation; appendages elongation |
| Copepoda |  | **Copepoda sp1** | Aquatic | 0 | 0 | 1 | 2 | Eyes absence; depigmentation; appendages elongation |
|  |  | **Nemertea sp1** | Terrestrial | 0 | 0 | 1 | 2 | Depigmentation; body elongarion |
|  |  | Pauropoda sp1 | Terrestrial | 0 | 0 | 1 | 2 |  |
|  |  | Symphyla sp1 | Terrestrial | 0 | 0 | 1 | 2 |  |
|  |  | **Symphyla sp2** | Terrestrial | 0 | 3 | 1 | 20 | Appendages elongation; developed sensory organs |
|  |  | Turbellaria sp2 | Terrestrial | 0 | 0 | 1 | 20 |  |
